# Acceptance as a possible link between past psychedelic experiences and psychological flexibility

**DOI:** 10.1038/s41598-024-75595-8

**Published:** 2024-10-16

**Authors:** Andreas Krabbe, Pilleriin Sikka, Jussi Jylkkä

**Affiliations:** 1 Department of Psychology, Åbo Akademi University, Arken Tehtaankatu 2, FI-20500 Turku, Finland; 2 Department of Psychology, University of Turku, Turku, Finland; 3 Department of Cognitive Neuroscience and Philosophy, University of Skövde, Skövde, Sweden; 4 Department of Psychology, Stanford University, Stanford, CA USA; 5 Department of Anesthesiology, Perioperative and Pain Medicine, Stanford University, Stanford, CA USA

**Keywords:** Psychological flexibility / inflexibility, Network analysis, Well-being, Psychedelics, Psychological insights, Psychology, Human behaviour

## Abstract

Increased psychological flexibility (PF) may underlie the lasting positive effects of psychedelic experiences on mental well-being. The associations between different components of PF, psychological inflexibility (PI), and well-being with psychedelic use are not well understood. We conducted a cross-sectional internet survey of participants (*N =* 629) with experience of classical psychedelics. Using network analysis, we examined how aspects of a single psychedelic experience (mystical-type features and psychological insights) and the frequency of past psychedelic use, were associated with current PF and PI components, as well as with mental well-being and ill-being. Mediation analyses explored whether PF mediated the relationship between past psychedelic use and well-being or ill-being. The network analysis linked psychological insight to the PF component Acceptance, with no association found between the frequency of past use and PF. Mediation analyses showed PF mediates the association between past psychedelic use and well-being and ill-being. These results suggest that the quality and depth of the psychedelic experience, rather than the frequency of use, are primarily linked to psychological flexibility, particularly Acceptance, and overall well-being. This underscores the importance of treating PF as a multidimensional construct to better understand the long-term mental health benefits of psychedelics.

**Supplementary Information:**

The online version contains supplementary material available at 10.1038/s41598-024-75595-8.

Classic serotonergic psychedelics, including substances such as psilocybin, lysergic acid diethylamide (LSD), N,N-dimethyltryptamine (DMT), and mescaline, have demonstrated a broad spectrum of positive effects on mental health and well-being in both clinical trials^1^ and naturalistic settings^2^. Recent research emphasises the importance of acute, psychologically transformative insight- and/or mystical-type experiences as predictors of improved health outcomes in psychedelic-assisted therapy^3–5^. Mystical-type experiences typically involve a sense of unity, feelings of sacredness, positive mood, and a feeling of connectedness with something divine or spiritual^6^. Psychological insights usually entail awareness of relationships, past events, goals, and values^4^. However, the psychological factors underlying post-acute, sustained changes in well-being remain poorly understood.

One factor that has been suggested to contribute to long-term therapeutic benefits in psychedelic-assisted therapy is increased psychological flexibility^4,7,8^. Psychological flexibility, as conceptualised by Hayes et al., refers to “the ability to contact the present moment more fully as a conscious human being, and to change or persist in behaviours when doing so serves valued ends”^9^. The Psychological flexibility construct encompasses six adaptive psychological components contributing to human flourishing: *Acceptance* (of private events such as thoughts and emotions), *Defusion* (from literal belief in one’s thoughts), *Present Moment Awareness*, *Self-as-Context* (viewing oneself as an observer of mental events), *Values* clarification, and *Committed Action* (maintaining behaviours aligned with personally relevant aspects of one’s life)^9^. Conversely, psychological inflexibility refers to how “language and cognition interact with direct contingencies to produce an inability to persist or change behaviour in the service of long-term valued ends”^9^. The psychological inflexibility construct comprises six maladaptive components: *Experiential Avoidance*, *Cognitive Fusion* (with internal events), *Lack of Present Moment Awareness*, *Self-as-Content* (strict adherence to conceptualisations of the self-and/or failure to see oneself as an observer), *Lack of Contact with Values*, and *Inaction or Impulsivity* (failure to behave in line with personal values)^9^. While these six core components constituting psychological flexibility and inflexibility, respectively, are regarded as independent, they are also interconnected because the components affect each other in a reciprocal manner. That is, while each component can be targeted on its own during interventions, the activation of one component usually leads to changes in other components^9^.

Preliminary findings suggest that psychedelic experiences are positively associated with components of psychological flexibility. For instance, a qualitative study on psilocybin-assisted therapy for depression revealed that psilocybin encouraged connection and acceptance^10^. Others reported an enhanced sense of connectedness to values and better adherence to value congruent behaviour following psychedelic experiences^11^. Recently, the principles of Acceptance and Commitment Therapy (ACT), emphasising psychological flexibility as a primary treatment target, have been introduced as the main therapeutic modality in psilocybin-assisted therapy for depression^12^. Theoretical work based on clinical observations of psilocybin-assisted therapy has led to new ways of conceptualising how psychological flexibility components relate to each other. According to the Accept, Connect, Embody (ACE) model, psychedelics can foster better acceptance of difficult emotions and private content, which in turn can lead to improved connectedness with values^13^. Nevertheless, empirical research on how psychedelic experiences relate to different psychological flexibility components remains limited.

Regarding empirical research on psychological inflexibility and its association with ill-being, a recent cross-sectional study showed that psychological insights experienced during the acute phase of a psychedelic experience were associated with retrospectively measured decreased psychological inflexibility, which in turn mediated decreases in depression and anxiety symptoms^8^. Similarly, a longitudinal study demonstrated that decreased psychological inflexibility resulting from psychedelic experiences correlated with reduced symptoms of depression^7^. Decreased psychological inflexibility has also been identified as a mediator in the connection between psychedelic experiences and a decrease in symptoms of PTSD resulting from continuous experiences of racial discrimination among black people, indigenous people, and people of colour^14^. A study that explicitly addressed well-being found that decreased psychological inflexibility mediated increases in positive mood and cognitive reappraisal in ceremonial Ayahuasca users^15^. Others found that psychological flexibility, multiple facets of mindfulness, and value-congruent living significantly improved following psilocybin-assisted therapy and were sustained during the 16 weeks after the intervention. Moreover, increases in psychological flexibility and experiential acceptance were strongly linked to reductions in depression severity after psilocybin use^16^. A recent pilot study of nine participants attending a 7-day psilocybin retreat found increases in cognitive defusion, valued living, self-compassion and several components of psychological flexibility and other outcome measures relating to well-being. Furthermore, participants showed a reduction in psychological inflexibility^17^.

Most research on psychological flexibility concerning psychedelic experiences has treated it as a unified construct, primarily relying on the Acceptance and Action Questionnaire (AAQ/AAQ-II)^7,8,14,15^. However, the AAQ-II mainly measures experiential avoidance rather than other components of psychological flexibility / inflexibility, and its correlation with psychological distress has raised validity concerns^18,19^. Additionally, considering psychological flexibility and inflexibility as opposite ends of a continuum may be an oversimplification, as individuals can exhibit various combinations of psychological flexibility and inflexibility components simultaneously^20–22^. Newer instruments such as the Multidimensional Psychological Flexibility Inventory (MPFI)^23^ offer a more comprehensive assessment, capturing all twelve components of psychological flexibility and inflexibility.

Lastly, researchers recommend using modelling methods that address multicollinearity between components when examining interconnected multicomponential constructs such as psychological flexibility and inflexibility^24–27^. Network analysis, a valuable tool in this context, can be used to explore structural relations between components and their associations with important outcome variables^28–30^. In psychological network analysis, constructs are represented as interconnected nodes, with edges indicating the degree of association between nodes. Networks can be estimated using many different methods; however, the most common method involves the estimation of partial correlation coefficients from covariance matrices^29^. Nevertheless, this method has not been used in psychedelic research, although it is well suited for exploring complex phenomena such as psychedelic experiences and their possible aftereffects on multicomponential constructs such as psychological flexibility and inflexibility.

## Aims of the present study

Previous psychedelic research has mainly treated psychological flexibility and inflexibility as unitary constructs (e.g., Refs.^7,8,14^). However, both psychological flexibility and inflexibility consist of six interconnected components^9^. Therefore, in the present study, we used network analysis to measure how participants’ past experiences with psychedelics is associated with different components of present moment psychological flexibility / inflexibility as well as with mental well-being and ill-being. Two network models were used. One model (“Acute Model”) included the measures of acute features of a single, personally meaningful psychedelic experience in the participants’ past (i.e., psychological insight and mystical-type features); the other model (“Frequency Model”) included measures relating to the frequency of past psychedelic use. In addition to network analysis, we implemented mediation analysis to examine whether possible links between past psychedelic experiences and well-being and ill-being are mediated by psychological flexibility.

## Method

### Participants and procedure

Out of 2500 participants who completed a pre-screening survey aimed at identifying prior psychedelic experience, 840 were eligible for the main survey; that is, they were at least 18 years of age and had had at least one meaningful experience facilitated by a classical psychedelic substance. From this group, *N* = 701 participants completed the survey. After exclusions − 14 who reported having zero lifetime experience with classical psychedelics (and therefore probably misunderstood the survey instructions), 29 whose experience was facilitated by other drug(s) than the specified psychedelics, 16 who used more than two psychedelics simultaneously to facilitate the experience, and 13 who used additional drugs such as cannabis-thus the final sample comprised *N* = 629 participants.

Participants were recruited via the online platform Prolific^27^, where they received an anonymous participant ID and were directed to the in-house survey platform “Soile”. Before accessing the survey, participants completed an informed consent form outlining the study’s purpose and participation criteria (18 years or older, prior experience with classical psychedelics such as LSD, psilocybin, Ayahuasca, DMT, 5-MeO-DMT, or mescaline). The main survey included demographic questions (age, gender, socioeconomic status), details about past psychedelic use (types of psychedelics, doses, frequency), scales measuring well-being and ill-being, and inquiries about a single meaningful past psychedelic experience. It also included scales for assessing worldviews and metaphysical beliefs, which were analysed separately and reported in^31^.

The data was collected in accordance with GDPR regulations and was conducted in accordance with the Declaration of Helsinki. The study was approved by the Ethics Board of the Departments of Psychology and Logopaedics at Åbo Akademi University, Finland (decision number 22/2022). The studies (Studies 1a and 1b) were pre-registered at (https://osf.io/pbcvq).

## Measures

### Features of the psychedelic experience

The Mystical Experience Questionnaire (MEQ30)^3^ was used to measure mystical-type phenomena during a personally meaningful past psychedelic experience. The MEQ30 comprises 30 items across four factors: mystical, positive mood, transcendence of time and space, and ineffability. Ratings were given on a six-point Likert scale ranging from 0 (“None at all”) to 5 (“Extreme (more than ever before in my life)”), and mean scores were calculated to measure the overall degree of mystical-type experiences, with higher scores indicating a greater intensity of these experiences.

The Psychological Insight Questionnaire (PIQ)^4^ was used to measure participants’ psychological insights during a personally meaningful past psychedelic experience. Each of the 28 items was rated on a six-point Likert scale from 0 (“None at all”) to 5 (“Extreme (more than ever before in my life)”), with scores reflecting the degree of insights into Avoidance and Maladaptive Patterns (AMP) and Goals and Adaptive Patterns (GAP). Mean scores, as well as subscale scores for AMP and GAP, were calculated, with higher values indicating a greater degree of psychological insight experiences.

The frequency of past psychedelic use was assessed by asking the participants about the total number of times they had used psychedelics during their life (“cumulative frequency”), the duration since their last experience in months (“time since last use”), their typical frequency of psychedelic use (“average use”), and their average dose (“average dose”). The “average use” was quantified on a scale ranging from “Never” (0) to “Daily” (8), while “average dose” was rated on a three-point scale from “small” (1) to “strong” (3) or “I don’t know,” with reference to typical doses of commonly used substances.

### Psychological flexibility and inflexibility

The Multidimensional Psychological Flexibility Inventory (MPFI-24)^23^ was used to assess psychological flexibility and inflexibility. The MPFI consists of 24 items assessing the six psychological components constituting psychological flexibility (Acceptance, Present Moment Awareness, Self-as-Context, Defusion, Values, Committed Action), and the six components constituting inflexibility (Experiential Avoidance, Lack of Awareness, Self-as-Content, Fusion, Lack of Contact with Values, Inaction). Participants rated the frequency of each item over the past two weeks on a six-point Likert scale from 1 (“Never true”) to 6 (“Always true”). The mean scores of the twelve subscales were calculated, with higher scores reflecting a greater degree of psychological flexibility or inflexibility, respectively.

### Well-being

The Warwick-Edinburgh Mental Wellbeing Scale (WEMWBS)^32^ was used to measure mental well-being. The scale consists of 14 items addressing different aspects of mental well-being. Participants rated how often they experienced each item during the past two weeks on a five-point Likert scale ranging from 1 (“None of the time”) to 5 (“All of the time”). The item scores were averaged to produce a single score, with higher scores reflecting greater level of mental well-being.

The Peace of Mind Scale (PoMS)^33^ was used to assess how frequently participants experienced internal peace and harmony in their lives. The PoMS was originally developed to measure happiness in a Chinese cultural context, where more value is placed on inner harmony as opposed to “high arousal positive affect” states (e.g., feeling excited, elated); however, the scale has also been shown to work well in a Western cultural context^34–36^. The PoMS consists of 7 items rated on a five-point Likert scale ranging from 1 (“Not at all”) to 5 (“All of the time scale”). The items were averaged to produce a single score, with higher scores reflecting greater levels of peace of mind.

### Ill-being

Symptoms of depression were assessed with the depression module of the Patient Health Questionnaire (PHQ-9)^37^. The scale consists of nine items addressing each of the diagnostic criteria for depression from the Diagnostic and Statistical Manual of Mental Disorders (DSM-5; American Psychiatric Association, 2013). Participants rated how often, in the past two weeks, each of the nine symptoms bothered them on a scale ranging from 0 (“Not at all”) to 3 (“Nearly every day”). The items were averaged to produce a single score, with higher scores reflecting higher levels of symptoms of depression.

Symptoms of anxiety were assessed using the Generalized Anxiety Disorder Scale (GAD-7)^38^. The scale consists of 7 items that are based on diagnostic criteria A, B, and C for generalized anxiety disorder from the DSM-5; American Psychiatric Association, 2013). Participants rated how often, in the past two weeks, each of the symptoms bothered them on a scale ranging from 0 (“Not at all”) to 3 (“Nearly every day”). The items were averaged to produce a single score, with higher scores reflecting greater levels of symptoms of anxiety.

## Analytic approach

### Network analysis

 In the Acute model we performed a network analysis on mean scores from the MEQ30, PIQ, PoMS, WEMWBS, GAD-7, PHQ-9, and MPFI subscales, which represent the 12 components of psychological flexibility and inflexibility. This resulted in an 18-node network for the Acute model. In the Frequency model we replaced the acute measures with frequency measures, which resulted in a 19-node network. The average dose was omitted from the Frequency model because a large number of participants (*n* = 135) did not know the average dose. Using R (version 4.2.3), we performed network estimation, inference (including properties and centrality), stability checks, and exploratory graph analysis for community detection. All variables were assessed for univariate and multivariate normality using the *MVN* package^39^. The data deviated from multivariate normality based on Mardia’s skewness for the acute model (S = 2096.544, *p* < .001) and kurtosis (K = 19.666, *p* < .001) as well as for the frequency model (S = 21908.335, *p* < .001) and kurtosis (K = 119.017, *p* < .001). No responses included any missing data, (*N* = 629).

### Network estimation

We used the *mgm* package^40^ to estimate a Gaussian Graphical Model (GGM) partial correlation network and to obtain predictability or R2 estimates of each node in the network. Before estimation, we conducted nonparanormal transformations^41^ on the variables to achieve a marginal normal distribution^42^ using the *huge.npn* function from the *huge* package^43^. We used graphical LASSO^44^ in combination with EBIC model selection^45^ and a tuning parameter (gamma) set to 0.5 to regularise and optimise the specificity and sensitivity of the network. To graphically represent the results, we used the modified Fruchterman-Reingold algorithm^46^ for weighted networks implemented in the *qgraph* package^47^. We used the *bootnet* package^48^ to estimate the stability and robustness of the network parameters. To check the stability of the edges, we estimated non-parametric bootstrap 95% confidence intervals (CIs) on the edge weights with 1000 bootstrap samples, while we estimated the stability of the node centrality indices using the case-dropping bootstrap procedure with 2500 bootstrapped samples. To determine whether the components of psychological flexibility and inflexibility formed separate clusters, as suggested by earlier network studies and previous theoretical work on psychological flexibility^23,28,30^ we conducted exploratory graph analysis (EGA) using the Walktrap algorithm implemented in the *EGAnet* package^49^. We used the bootEGA algorithm^50^ to estimate the stability of the communities by performing non-parametric bootstrapped exploratory graph analysis using GLASSO regularisation with 1000 bootstrapped samples, utilising the Walktrap algorithm implemented in the *EGAnet* package.

### Mediation analysis

 The mediation analysis deviated from the preregistration. In our preregistration, we specified that we would use the same measures in the mediation analyses as in the network model. However, we decided to include only an aggregated measure of psychological flexibility, because most previous studies have used aggregate variables. This renders the results of the present study more comparable to existing research. Another reason for including only an aggregate measure of psychological flexibility (and conducting mediation analyses instead of a full SEM model) was to make the results clearer and easier to understand. This approach allowed us to provide broader view of associations with the mediation analyses and a more granular view of associations with the network models.

We conducted mediation analysis in JASP 0.17.1, using standardised estimates and bootstrap analyses with 1000 replications to obtain bias-corrected percentiles for our 95% CI estimates. The Acute model included the mean scores of MEQ and PIQ as predictors; psychological flexibility (total mean score) as mediator; and the PoMS, WEMWBS, GAD-7, and PHQ-9 scores as outcome variables. Age and gender were added as covariates. Due to the small number of participants reporting other as gender (*n =* 5), the mediation analyses included only males (*n =* 362) and females (*n =* 262). Gender was dummy coded as 0 for male and 1 for female. Standardised estimates were used for continuous variables. For the Frequency model we substituted the acute measures with frequency measures (cumulative frequency, time since last use, and average frequency). Otherwise, the variables in the models were the same. In both models the significance threshold was set to 0.05.

## Results

### Descriptive statistics

Demographics and information about past psychedelic use are presented in Table 1. Table 2 shows the mean scores and internal consistency of the scales as well as bivariate correlations between all the variables measured in the study.

**Table 1:** Demographics and past psychedelic use.

Demographic information	M	SD	Min	Max	n	%
Age	32.25	10.20	19.00	74.00		
Male					362	58.00
Female					262	42.00
Other					5	8.00
Education level						
Lower Secondary education					10	1.59
Upper Secondary or Vocational education					201	31.96
University: Bachelor’s degree					292	46.42
University: Master’s degree					120	19.08
University: Doctoral degree					6	0.95
Income level [1–5]	2.77	0.89	1.00	5.00		
Past psychedelic use						
Cumulative frequency	7.70	26.72	1.00	500.00		
Time since last use (years)	2.93	5.48	0.00	45.83		
Time since most meaningful experience (years)	2.22	3.50	0.00	25.00		
Average frequency of use						
Once every four years or less often					290	46.10
Once every three years					31	4.93
Once every two years					70	11.13
Once a year					81	12.88
Few times a year					128	20.35
Monthly					21	3.34
Weekly					6	0.95
Daily					2	0.32
Average dose [1–3]	1.42	0.58	1.00	3.00		
Experience facilitated by						
LSD					293	47.00
Psilocybin					339	54.00
DMT					40	6.00
5-MeO-DMT					4	0.60
Mescaline					20	3.00
Most frequently reported setting						
Home or home-like environment					440	70.00
Nature					56	9.00
Public gathering (e.g., a festival)					56	9.00
Most frequently reported intention						
To relax and enjoy					377	60.00
Curiosity					327	52.00
Spiritual					63	10.00
Therapeutic					44	7.00
Most frequently reported country of origin						
South Africa					150	24.00
UK					111	18.00
Poland					74	12.00
Portugal					53	8.5
Other					236	37.5

The average dose was estimated on an ordinal scale from 1 to 3 (1 = Small; 2 = Medium; 3 = Strong) with a “Don’t know” option included. Examples of typical doses of commonly used substances were given. *LSD* Lysergic acid diethylamide,* DMT* Dimethyltryptamine,* 5-MeO-DMT* 5-methoxy-N, N-dimethyltryptamine.

**Table 2:** Correlations and descriptive statistics of scales used in the study.

Variable	1	2	3	4	5	6	7	8	9	10	11	12	13	14	15	16	17	18	19	20	21	22	23	24	25
1. Acceptance	—																								
2. Awareness	0.38***	—																							
3. Self-as-Context	0.34***	0.52***	—																						
4. Defusion	0.21***	0.39***	0.53***	—																					
5. Values	0.29***	0.53***	0.56***	0.49***	—																				
6. Committed Action	0.23***	0.47***	0.53***	0.44***	0.60***	—																			
7. Flexibility Mean	0.54***	0.74***	0.79***	0.70***	0.79***	0.75***	—																		
8. Experiential Avoidance	0.09*	0.09*	0.13***	0.08	0.11**	0.11**	0.14***	—																	
9. Lack of Awareness	0.05	− 0.11**	− 0.11**	− 0.07	− 0.11**	− 0.14***	− 0.12**	0.14***	—																
10. Self-as-Content	0.13***	− 0.08*	− 0.06	− 0.18***	− 0.11**	− 0.16***	− 0.10**	0.20***	0.40***	—															
11. Fusion	0.12**	− 0.11**	− 0.19***	− 0.38***	− 0.19***	− 0.25***	− 0.23***	0.19***	0.38***	0.61***	—														
12. Lack of Values	0.08*	− 0.17***	− 0.13**	− 0.23***	− 0.22***	− 0.29***	− 0.21***	0.15***	0.39***	0.57***	0.62***	—													
13. Inaction	0.08*	− 0.15***	− 0.18***	− 0.31***	− 0.26***	− 0.36***	− 0.27***	0.08*	0.40***	0.54***	0.71***	0.66***	—												
14. Inflexibility Mean	0.13***	− 0.12**	− 0.13**	− 0.26***	− 0.18***	− 0.25***	− 0.18***	0.39***	0.62***	0.78***	0.83***	0.79***	0.8***	—											
15. WEMWBS	0.11**	0.36***	0.37***	0.47***	0.48***	0.50***	0.53***	− 0.01	− 0.26***	− 0.34***	− 0.55***	− 0.39***	− 0.55***	− 0.49***	—										
16. PoMS	0.03	0.26***	0.31***	0.45***	0.39***	0.40***	0.42***	− 0.06	− 0.30***	− 0.41***	− 0.59***	− 0.42***	− 0.54***	− 0.55***	0.78***	—									
17. GAD-7	0.10**	− 0.14***	− 0.19***	− 0.33***	− 0.28***	− 0.26***	− 0.25***	0.16***	0.32***	0.51***	0.64***	0.51***	0.61***	0.66***	− 0.59***	− 0.66***	—								
18. PHQ-9	0.10*	− 0.23***	− 0.23***	− 0.3***	− 0.32***	− 0.33***	− 0.29***	0.12**	0.39***	0.49***	0.61***	0.53***	0.62***	0.65***	− 0.64***	− 0.63***	0.81***	—							
19. MEQ-30	0.19***	0.21***	0.17***	0.15***	0.16***	0.20***	0.25***	0.12**	0.04	0.07	0.06	0.06	0.02	0.09*	0.12**	0.05	0.04	0.04	—						
20. PIQ	0.30***	0.23***	0.23***	0.19***	0.17***	0.16***	0.29***	0.13**	0.08*	0.16***	0.10*	0.17***	0.09*	0.17***	0.12**	0.05	0.15***	0.13***	0.60***	—					
21. AMP	0.29***	0.20***	0.20***	0.16***	0.13**	0.12**	0.25***	0.13**	0.09*	0.19***	0.11**	0.19***	0.11**	0.19***	0.07	− 0.01	0.19***	0.15***	0.52***	0.97***	—				
22. GAP	0.29***	0.24***	0.25***	0.21***	0.21***	0.22***	0.33***	0.12**	0.07	0.10**	0.06	0.11**	0.03	0.12***	0.18***	0.11**	0.08	0.09*	0.66***	0.94***	0.84***	—			
23. Average use	0.15***	0.07	0.12**	0.09*	0.01	0.06	0.11**	0.06	− 0.07	0.03	− 0.04	0.06	− 0.01	0.01	0.06	0.05	0.01	0.01	0.30***	0.41***	0.37***	0.42***	—		
24. Cum. Frequency	− 0.01	0.05	0.08	0.08	0.02	0.05	0.07	− 0.02	0.01	− 0.03	− 0.02	− 0.07	− 0.06	− 0.04	0.01	0.02	− 0.03	− 0.01	0.01	− 0.01	− 0.03	0.01	− 0.01	—	
25. Time since last use	− 0.02	− 0.04	− 0.04	− 0.03	− 0.02	− 0.05	− 0.06	0.02	0.06	− 0.01	0.05	0.03	0.03	0.05	− 0.05	− 0.04	0.06	0.01	− 0.04	0.01	0.01	− 0.02	0.02	− 0.29***	—
Mean	3.39	4.06	4.00	3.49	4.03	4.13	3.85	3.83	2.92	2.98	3.12	2.82	2.96	3.11	3.31	3.00	2.05	1.90	2.62	1.82	1.75	1.93	2.62	7.70	37.70
Standard deviation	1.02	1.02	1.09	1.16	1.11	1.07	0.79	1.21	1.20	1.22	1.27	1.15	1.29	0.88	0.71	0.88	0.77	0.70	0.94	1.09	1.11	1.16	1.93	26.72	78.18
Cronbah’s α	0.60	0.75	0.72	0.84	0.78	0.79	0.88	0.82	0.85	0.77	0.85	0.75	0.88	0.88	0.93	0.90	0.91	0.90	0.95	0.96	0.94	0.92			

Variables 1–14 represent means of psychological flexibility and inflexibility as well as subscales of the Multidimensional Psychological Flexibility Inventory (MPFI; Range: 1–6), Patient Health Questionnaire (PHQ-9; Range: 0–3), Generalized Anxiety Disorder Scale (GAD-7; Range: 0–3), The Peace of Mind Scale (PoMS; Range: 1–5), Warwick-Edinburgh Mental Wellbeing Scale (WEMWBS; Range: 1–5).

The Psychological Insight Questionnaire (PIQ; Range: 0–5), The Mystical Experience Questionnaire (MEQ30; Range: 0–5). Avoidance and Maladaptive Patterns Insights (AMP) subscale, Goals and Adaptive Patterns Insights (GAP) subscale. *p < .05. **p < .01. ***p < .001..

### Network properties and associations

**Fig. 1:**
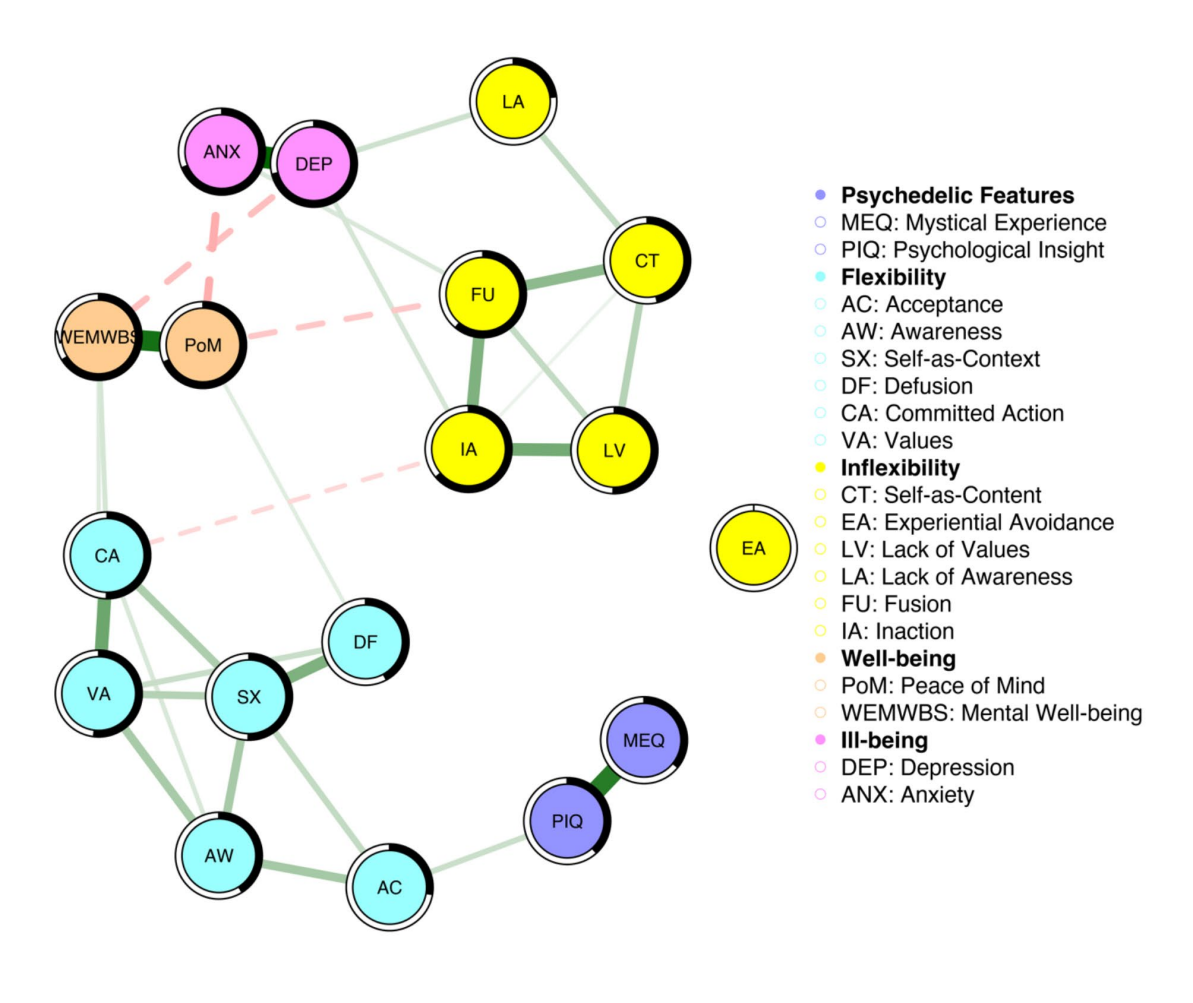
Figure represents the Acute model. The colour intensity and width of each edge reflect the relative strength of the respective associations (partial correlations), and the colour and line-type of the edges indicate the direction of the associations (green-solid = positive; red-dashed = negative). The black shading encompassing the nodes represents the proportion of variance or R2 accounted for by the respective nodes within the network.

In the Acute model, nodes representing psychological flexibility components formed a cohesive and distinct cluster. Similarly, components of psychological inflexibility formed a distinct cluster, except for Experiential Avoidance, which was not associated with other psychological inflexibility nodes. Strong associations were observed within the psychological flexibility and inflexibility clusters, while inter-cluster associations were comparatively weaker. Figure 1 illustrates the network’s structure and associations among measures, encompassing features of a past psychedelic experience (mystical-type and psychological insight), psychological flexibility / inflexibility, and well-being/ill-being.

Nodes representing mystical-type features and psychological insight showed a strong positive association (0.47). Psychological insight was the only node representing the features of psychedelic experience that was clearly associated with nodes representing psychological flexibility, specifically Acceptance (0.11). Nodes representing well-being and ill-being exhibited strong internal positive associations. Peace of mind was positively linked with mental well-being (0.51), and symptoms of depression were associated with symptoms of anxiety (0.55). Mental well-being was inversely correlated with depression (-0.14), while peace of mind was inversely correlated with symptoms of anxiety (-0.18). Regarding psychological flexibility and inflexibility, mental well-being was positively associated with Committed Action (0.09) and Values (0.07), while peace of mind was associated with Defusion (0.06) and inversely associated with Fusion (-0.12). Symptoms of anxiety were linked to Fusion (0.08), and symptoms of depression were associated with Lack of Awareness (0.11) and Inaction (0.09). Table S1 in the Appendix shows the LASSO-regularised connectivity coefficients between features of the psychedelic experience, psychological flexibility / inflexibility components and mental well-being and ill-being.

**Fig. 2:**
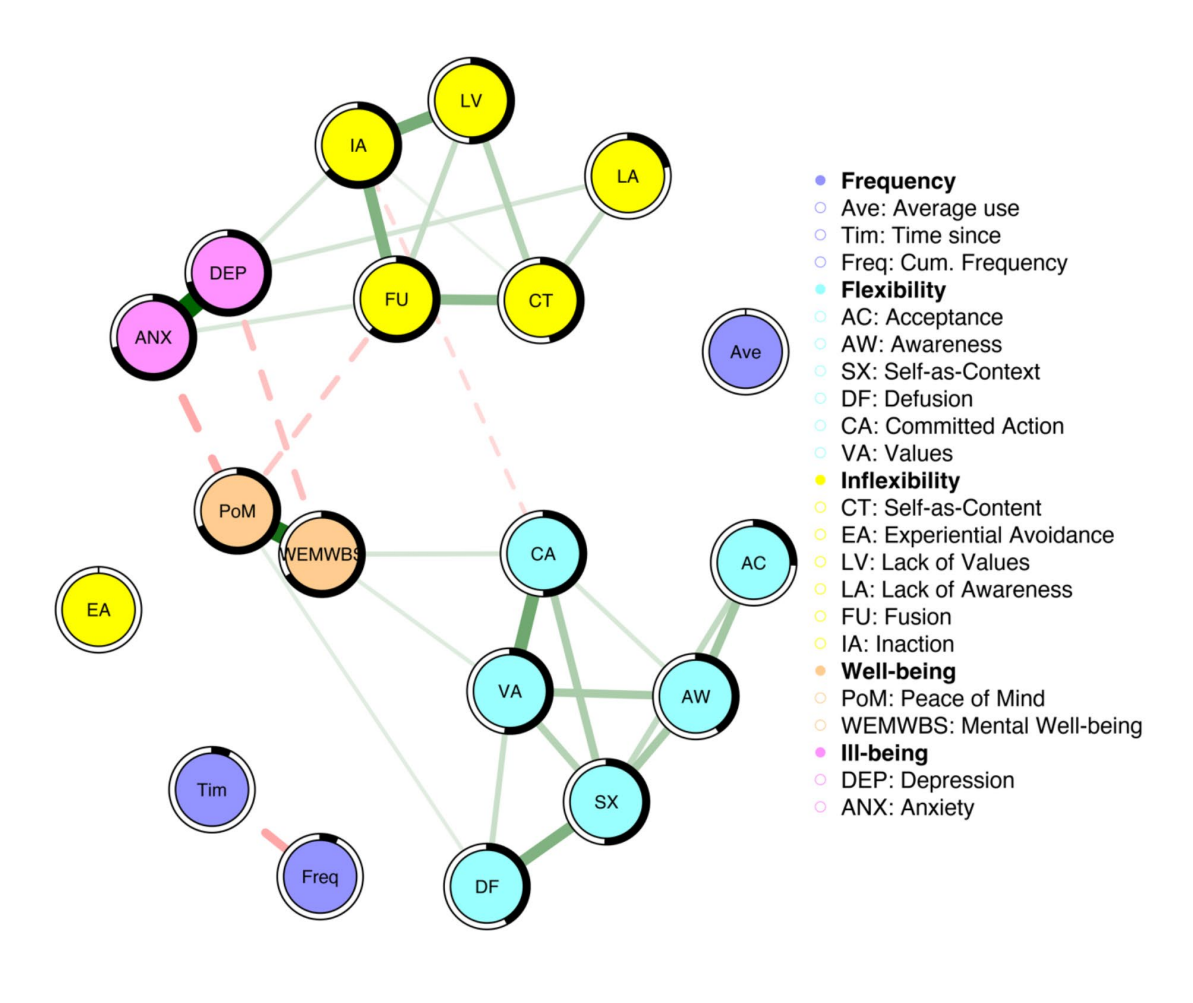
Figure represents the Frequency Model. The colour intensity and width of each edge reflect the relative strength of the respective associations (partial correlations), and the colour and line-type of the edges indicate the direction of the associations (green-solid = positive; red-dashed = negative). The black shading encompassing the nodes represents the proportion of variance or R2 accounted for by the respective nodes within the network.

For the Frequency Model, nodes representing psychological flexibility and inflexibility components formed cohesive and distinct clusters, except for Experiential Avoidance, which was not associated with other inflexibility nodes, in line with the Acute model. Strong associations were observed within the psychological flexibility and inflexibility clusters, while inter-cluster associations were comparatively weaker. Figure 2 illustrates the network’s structure and associations among measures, encompassing frequency measures relating to past psychedelic experiences, psychological flexibility / inflexibility components, and mental well-being and ill-being.

The nodes representing cumulative frequency and time since last use were inversely correlated (-0.20). Average frequency of use did not show any associations with the other frequency measures. Furthermore, cumulative frequency and time since last use did not show any association with other nodes. Nodes representing well-being and ill-being exhibited strong internal positive associations. Peace of mind was positively linked with mental well-being (0.51), and symptoms of depression were associated with symptoms of anxiety (0.57). Mental well-being was inversely correlated with depression (-0.14), while peace of mind was inversely correlated with symptoms of anxiety (-0.19). For psychological flexibility and inflexibility, mental well-being was positively associated with Committed Action (0.09 and Values (0.07), while peace of mind was associated with Defusion (0.06) and inversely associated with Fusion (-0.12). Symptoms of anxiety were linked to Fusion (0.09), and symptoms of depression were associated with Lack of Awareness (0.09) and Inaction (0.09). Table S2 in the Appendix shows the LASSO-regularised connectivity coefficients between frequency measures of past psychedelic experiences, psychological flexibility/inflexibility components and mental well-being and ill-being.

### Strength centrality estimates

To quantify how well the nodes were directly connected to other nodes in the network, centrality measures were estimated. Table S3 in the Appendix presents the raw and standardised centrality estimates for node connectedness in the Acute Model. The six most central nodes in the Acute Model, in descending order of raw estimates, indexing different components of psychological flexibility / inflexibility were Self-as-Context (0.93), Cognitive Fusion (0.85), Values (0.85), Inaction (0.80), Committed Action (0.75), and Acceptance (0.68). Regarding well-being and ill-being, the most central nodes were depression (0.89), peace of mind (0.87), anxiety (0.81) and mental well-being (0.81). Among the nodes representing features of past psychedelic experiences, psychological insight (0.58) was more central than mystical-type experience (0.47). In the Frequency Model, the six most central nodes indexing different components of psychological flexibility / inflexibility were Self-as-Context (0.95), Cognitive Fusion (0.86), Values (0.85), Inaction (0.80), Committed Action (0.76), and Awareness (0.67). As for well-being and ill-being, the most central nodes were depression (0.89), peace of mind (0.86), anxiety (0.84) and mental well-being (0.81). Among the measures representing the frequency of past psychedelic experiences, cumulative frequency of use (0.20) and time since last use (0.20) were the only measures that survived LASSO regularisation. See Table S4 in the Appendix for full raw and standardised centrality estimates for the Frequency Model.

### Network stability

To estimate the robustness of the network parameters, bootstrapped 95% CIs were estimated for edge weights. The bootstrapped edge weights closely followed the estimated edge weights in both the Acute and Frequency Model, and the results pointed to relatively narrow CIs, indicating robust results (see Fig. S1 and S2 in the Appendix). As for strength centrality, a correlation stability coefficient (CS) of 0.75 was estimated for both the Acute and Frequency model, which exceeded the recommended threshold of 0.5 for psychological networks and points to stable strength centrality estimates^48^. Figures S3 and S4 in the Appendix show case-drop bootstrap plots for the reported centrality indices for the Acute and Frequency Model respectively.

### Exploratory graph analysis

**Fig. 3:**
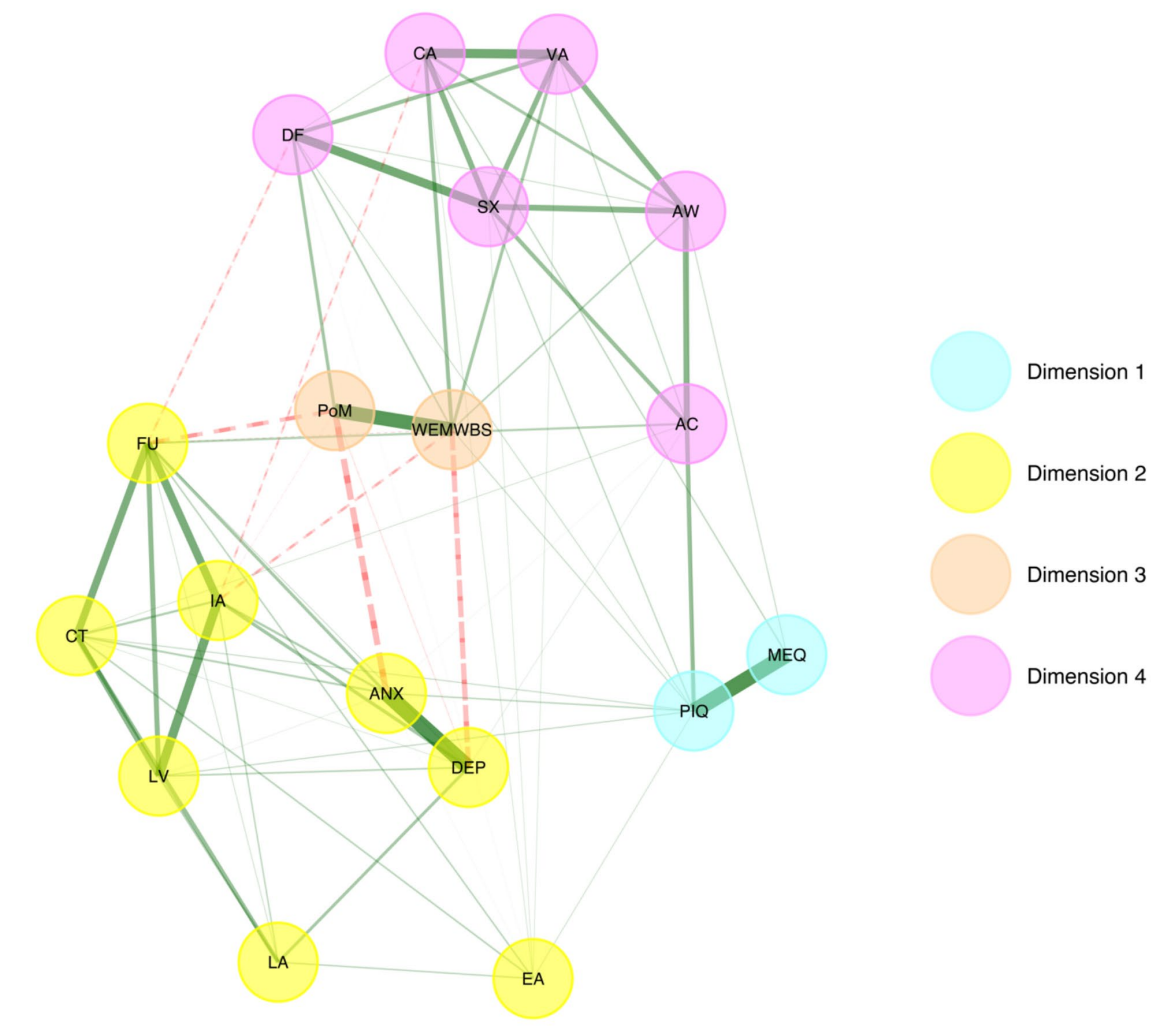
Results of Exploratory graph analysis (EGA) for the Acute Model. The turquoise dimension #1 = includes nodes indexing acute measures of a single past psychedelic experience. The yellow dimension #2 = includes nodes indexing psychological inflexibility components and ill-being. The orange dimension #3 = includes nodes indexing well-being. The magenta dimension #4 = includes psychological flexibility components. ANX = anxiety, DEP = depression, FU = fusion, CT = self-as-content, LA = lack of awareness, IA = inaction, LV = lack of values, EA = experiential avoidance, PoM = peace of mind, WEMWBS = mental well-being, CA = committed action, DF = defusion, VA = values, SX = self-as-context, AW = awareness, AC = acceptance, PIQ = psychological insight, MEQ = mystical-type experience. The colour and line type of the edges indicate the direction of the associations (green-solid = positive; red-dashed = negative).

To examine whether components of psychological flexibility and inflexibility formed separate clusters, as suggested by earlier network studies and previous theoretical work on psychological flexibility, exploratory graph analysis (EGA) was carried out. Findings from the EGA for the Acute Model resulted in four communities or clusters: (1) psychological flexibility, (2) features of past psychedelic experiences (mystical-type features, psychological insight), (3) well-being (mental well-being, peace of mind), and (4) ill-being (depression, anxiety) and psychological inflexibility. That is, ill-being and psychological inflexibility did not form distinct clusters (see Fig. 3).

**Fig. 4:**
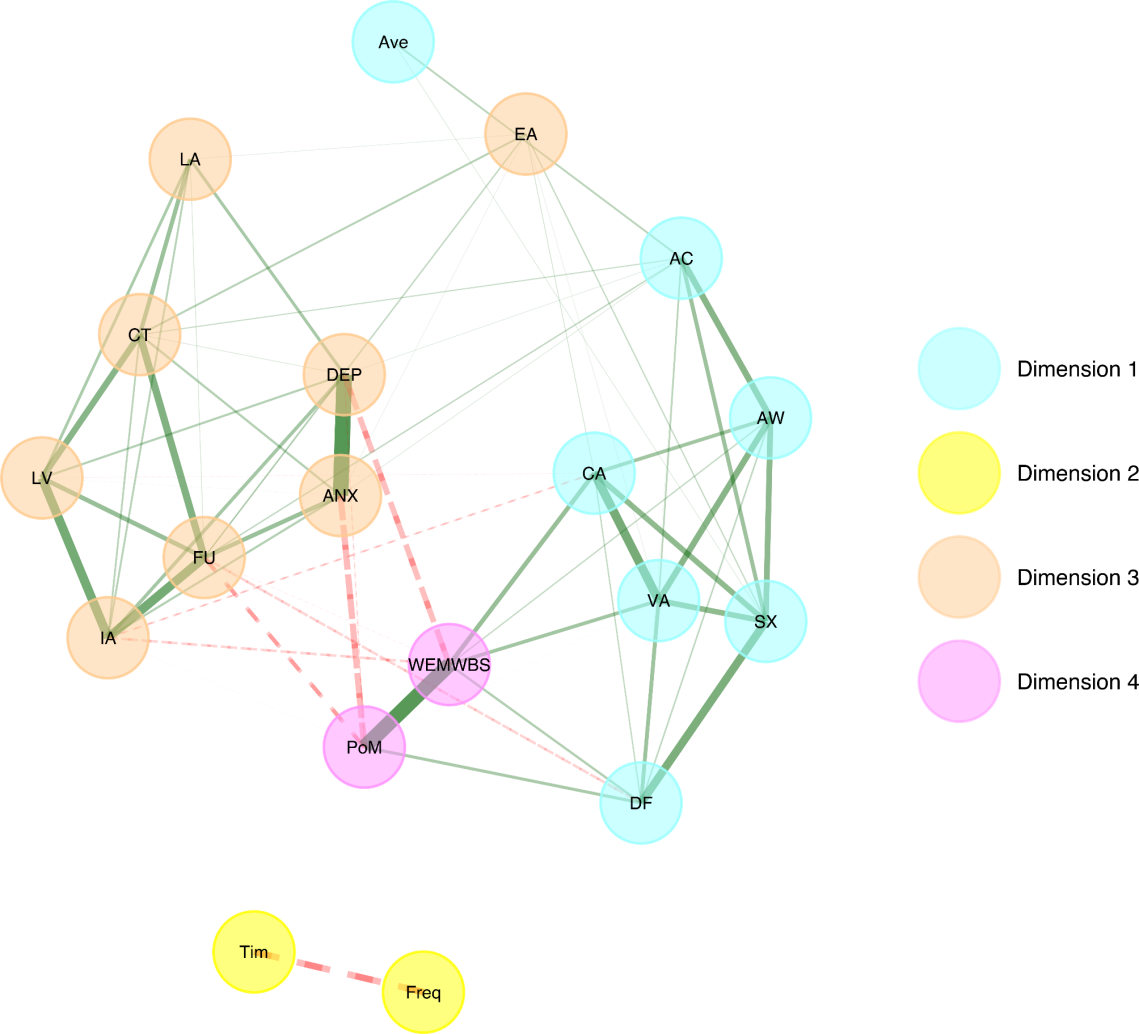
Results of Exploratory graph analysis (EGA) for the Frequency Model. The turquoise dimension #1 = includes nodes indexing psychological flexibility components and average psychedelic use frequency. The yellow dimension #2 = includes nodes indexing frequency measures, time since last use and cumulative frequency. The orange dimension #3 = includes nodes indexing psychological inflexibility components and ill-being. The magenta dimension #4 = includes nodes indexing well-being. ANX = anxiety, DEP = depression, FU = fusion, CT = self-as-content, LA = lack of awareness, IA = inaction, LV = lack of values, EA = experiential avoidance, PoM = peace of mind, WEMWBS = mental well-being, CA = committed action, DF = defusion, VA = values, SX = self-as-context, AW = awareness, AC = acceptance, Ave = average frequency of psychedelic use, Tim = time since last use, Freq = cumulative frequency. The colour and line type of the edges indicate the direction of the associations (green-solid = positive; red-dashed = negative).

The EGA for the Frequency Model also resulted in four clusters: (1) psychological flexibility and average use, (2) cumulative frequency and time since last use, (3) well-being (mental well-being, peace of mind), and (4) ill-being (depression, anxiety) and psychological inflexibility. That is, in the frequency model, ill-being and psychological inflexibility did not form separate clusters, in line with the Acute Model, and average use and psychological flexibility were also grouped into the same cluster (see Fig. 4).

To assess the stability of the clusters, we applied bootstrapped EGA using, non-parametric resampling method, GLASSO regularisation, and the Walktrap community detection algorithm with 1000 iterations. The full results of the bootstrapped EGA can be found in the Appendix. Overall, the bootstrapped EGA results for both the Frequency and the Acute Models suggested similar median partial correlation networks as the original EGAs, with a four-cluster solution identified ca. 89% and 85% of the time for the acute and frequency models, respectively (see Tables S5 and S6 in the Appendix). A further investigation of item stability, that is, how often the different nodes made up of mean scores, were assigned within certain clusters suggested that the nodes indexing ill-being, well-being and Experiential Avoidance were unstable, being assigned to their respective clusters only between 48% and 61% of the time in the Acute and Frequency Models. Average use was assigned to the psychological flexibility cluster approximately 70% of the time in the Frequency Model (see Figures S5 and S6). Results regarding the clustering of nodes relating to ill-being, and Experiential Avoidance as well as average use and components of psychological flexibility, should therefore be interpreted with some caution.

### Mediation analyses

**Fig. 5:**
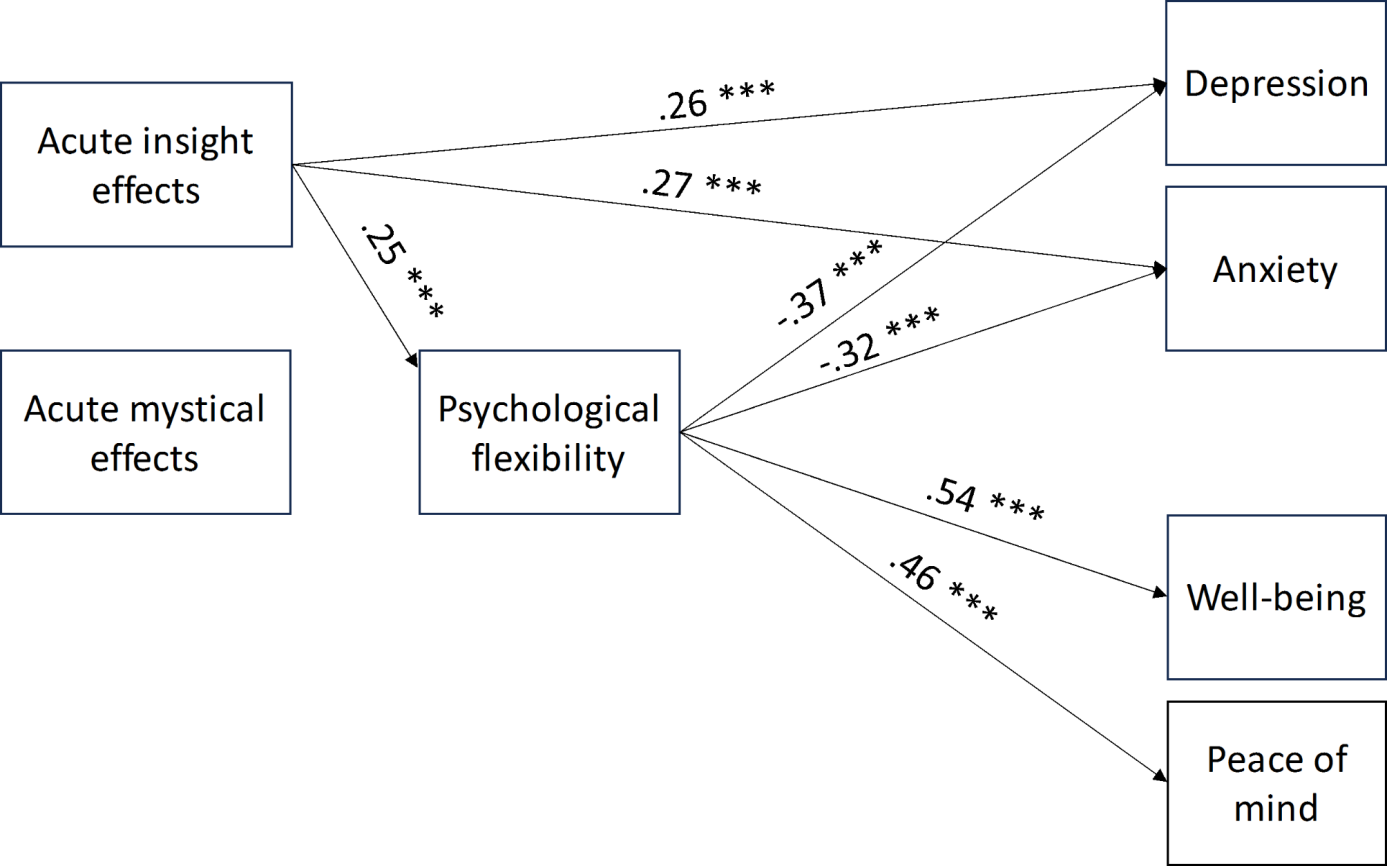
Mediation model with acute features of a meaningful past psychedelic experience as predictors. Only significant path coefficients are displayed.

The mediation analysis for the Acute Model included mean scores of MEQ30 and PIQ as predictors for indexing acute mystical-type and psychological insight experiences; psychological flexibility (total mean score) as mediator; and mean scores of PoMS, WEMWBS, GAD-7, and PHQ-9 as outcome variables indexing peace of mind, mental well-being, anxiety, and depression, respectively. Age and gender were added as covariates. The significance threshold was set at 0.05. No significant effects were found for acute mystical-type experiences. There was a significant direct effect of acute psychological insight experiences on depression (*E* = 0.26, *SE* = 0.05, *p* < .001) and anxiety (*E* = 0.27, *SE* = 0.05, *p* < .001). A significant indirect effect of psychological insight, mediated through psychological flexibility, was found for depression (*E* = − 0.09, *SE* = 0.02, *p* < .001), anxiety (*E* = − 0.08, *SE* = 0.02, *p* < .001), mental well-being (*E* = 0.14, *SE* = 0.03, *p* < .001), and peace of mind (*E* = 0.12, *SE* = 0.02, *p* = < 0.001). Age was weakly associated with psychological flexibility (*E* = 0.01, *SE* = 0.00, *p* = .05). Figure 5 shows significant path coefficients of the mediation analysis.

Given that the results from the mediation pointed to a direct positive association between acute psychological insights and depression/anxiety, a post hoc mediation analysis was conducted to examine whether the association was specifically related to one of the two subscales of the PIQ. The model included the two subscales of the PIQ indexing, Avoidance and Maladaptive Patterns (AMP) and Goals and Adaptive Patterns (GAP), as predictors; psychological flexibility as a mediator; and PoMS, WEMWBS, GAD-7, and PHQ-9 scores, which index peace of mind, mental well-being, anxiety, and depression, respectively, as outcome variables. Age and gender were added as covariates. The significance threshold was set at 0.05. The Goals and adaptive patterns subscale predicted mental well-being and peace of mind positively (*E* = 0.23, *SE* = 0.06, *p* < .001 and *E* = 0.24, *SE* = 0.07, *p* < .001) and anxiety negatively (*E* = − 0.16, *SE* = 0.07, *p* = .02). However, avoidance and maladaptive patterns negatively predicted mental well-being and peace of mind (*E* = − 0.23, *SE* = 0.06, *p* < .001 and *E* = − 0.30, *SE* = 0.07, *p* < .001), and depression and anxiety positively (*E* = 0.27, *SE* = 0.07, *p* < .001 and *E* = 0.40, *SE* = 0.07, *p* < .001). Significant indirect effects of goals and adaptive patterns through psychological flexibility were found for mental well-being (*E* = 0.21, *SE* = 0.04, *p* < .001), peace of mind (*E* = 0.17, *SE* = 0.03, *p* < .001), depression (*E* = − 0.14, *SE* = 0.03, *p* < .001), and anxiety (*E* = − 0.12, *SE* = 0.03, *p* < .001). There were no significant indirect effects of avoidance or maladaptive patterns through psychological flexibility on any of the outcome variables. Age was weakly associated with psychological flexibility (*E* = 0.01, *SE* = 0.00, *p* = .02) and avoidance and maladaptive patterns (*E* = 0.01, *SE* = 0.00, *p* = .05).

**Fig. 6:**
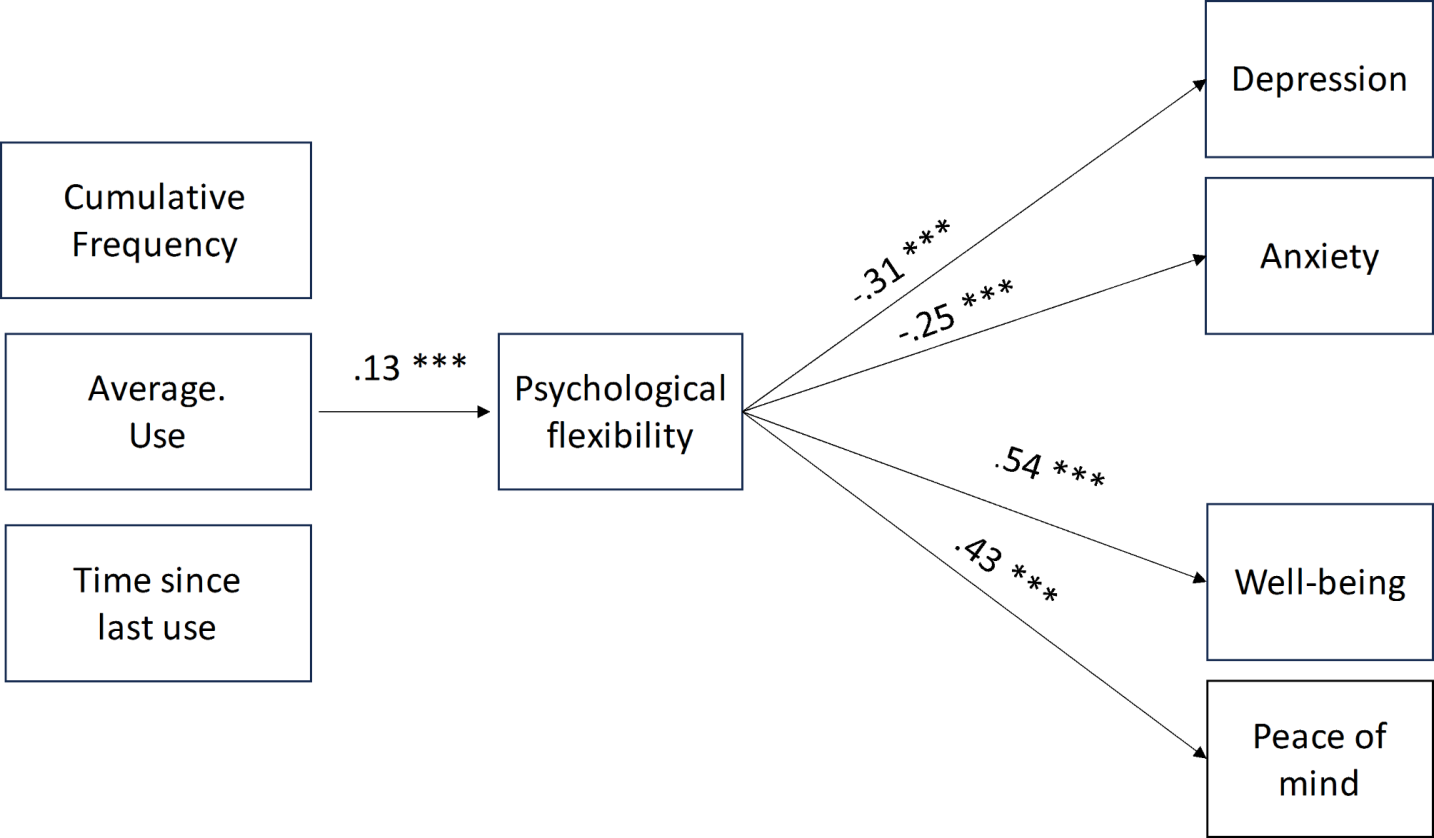
Mediation model with frequency measures relating to past psychedelic experiences as predictors. Only significant path coefficients are displayed.

The mediation analysis for the Frequency Model included mean scores of cumulative frequency, time since last use, and average frequency of psychedelic use as predictors; psychological flexibility (total mean score) as mediator; and mean scores of PoMS, WEMWBS, GAD-7, and PHQ-9 indexing peace of mind, mental well-being, anxiety, and depression, respectively, as outcome variables. Age and gender were added as covariates. The significance threshold was set at 0.05. No significant direct effects were found for any of the frequency measures. A significant indirect effect of average frequency of use, mediated through psychological flexibility, was found for depression (*E* = − 0.04, *SE* = 0.01, *p* < .001), anxiety (*E* = − 0.03, *SE* = 0.01, *p* < .001), mental well-being (*E* = 0.07, *SE* = 0.02, *p* < .001), and peace of mind (*E* = 0.06, *SE* = 0.02, *p* = < 0.001). Age was weakly associated with psychological flexibility (*E* = 0.01, *SE* = 0.00, *p* = .03) and cumulative frequency of use (*E* = 0.01, *SE* = 0.00, *p* = .03). Figure 6 shows significant path coefficients of the mediation analysis.

## Discussion

Studies investigating the possible psychological mechanisms underlying the post-acute effects of psychedelic experiences have suggested that increased psychological flexibility might be central to experiencing therapeutic benefits^7,8,16,17^. Earlier studies have typically treated psychological flexibility as a unitary construct. However, psychological flexibility and inflexibility are multicomponential constructs that both consist of six interrelated components. In this study, we used network analysis to explore how past psychedelic experiences, assessed retrospectively with acute measures indexing mystical-type and psychological insight experiences, are linked to various components of present moment psychological flexibility and inflexibility, as well as to well-being and ill-being. We also conducted the same analysis with frequency measures of psychedelic use. Moreover, we investigated whether psychological flexibility mediates the link between the acute aspects of a significant past psychedelic experience, the frequency of psychedelic use and the participants’ current well-being and ill-being.

### Network analysis of associations between past psychedelic experiences, psychological flexibility, and inflexibility

Acceptance was the only component of psychological flexibility that was clearly associated with the features of a past meaningful psychedelic experience when controlling for other components. However, this association applied to psychological insights only but not to mystical-type features of the experience. Earlier research has found stronger negative associations between psychological inflexibility and psychological insight than between psychological inflexibility and mystical-type experiences^8^. However, in the present study, no link was found between psychological inflexibility and psychological insight when controlling for other components.

The association between Acceptance and psychological insight aligns with the fundamental principle of psychedelic therapy, emphasising “learning to let go” or accepting private content arising during sessions^51^. Acceptance and commitment therapy (ACT), often employed in psychedelic-assisted psychotherapy, underscores acceptance over altering private experiences through mindfulness-based exercises^9^. Acceptance-based cognitive-behavioural therapies are prevalent in psychedelic-assisted psychotherapy due to their non-directive nature^12,13,52^. Narrative reports and clinical trials further support this, highlighting the importance of gaining insights into emotional or behavioural patterns and accepting them as part of one’s being for positive therapeutic outcomes^10,53^. Similar studies indicate that Acceptance, coupled with psychological insights, contributes to enduring positive outcomes, including enhanced body acceptance and coping with challenging life situations^53,54^. Lastly, increases in experiential acceptance following psilocybin-assisted treatment have been linked to decreases in depression severity^16^.

The cross-sectional design limits any causal inference between psychological insight and acceptance in this study. Furthermore, studies have shown that people with high trait acceptance experience more positive acute effects and fewer adverse reactions to psychedelics (Aday et al., 2021). Irrespective of the causal direction, the centrality of Acceptance as a key therapeutic process in the present study aligns with both Acceptance and Commitment Therapy (ACT) and earlier psychedelic studies^12,13,16,51,52^.

Regarding the Frequency Model, no direct association between past psychedelic use and psychological flexibility was found. Time since last use and cumulative frequency were strongly inversely associated, but average frequency did not show any association with other frequency measures or psychological flexibility/inflexibility components. Average frequency of use did correlate with the psychological flexibility components Acceptance (0.15), Self-as-context (0.12) and Defusion (0.09) at the bivariate level however, the correlations were weak.

Self-as-Context emerged as a highly central node, uniquely connecting with all the other components within the psychological flexibility cluster, consistent with findings from previous network studies^30^. This suggests that the ability to adopt a flexible, perspective-taking self is associated with all the other psychological flexibility components. In ACT theory, Self-as-context is considered foundational, ruling over and facilitating other psychological flexibility components^55^. Described as the capacity to observe feelings and thoughts from a detached perspective, Self-as-context aids in calmly and flexibly managing negative thoughts^9^. Low levels of Self-as-context have been associated with increased depression and anxiety^56–58^. Changes in the sense of self, alternatively referred to as ego dissolution or oceanic boundlessness, have been linked to successful outcomes in psychedelic-assisted therapy^59^. Theoretical perspectives highlight the shift from a judgmental self-narrative or ego to a more expansive, perspective-taking self as crucial for successful therapeutic outcomes in both ACT and psychedelic therapy^13,55^. Although Self-as-context correlated with psychological insights (0.23) at the bivariate level (the second highest after Acceptance), no direct association between Self-as-context and psychological insights was observed after controlling for other psychological flexibility components. Regarding the Frequency Model, no association was found between Self-as-context and past psychedelic use. However, similarly as in the Acute Model, Self-as-context was weakly associated with average use (0.12) at the bivariate level.

Two key components of psychological flexibility, Committed Action and Values, stand out as strongly associated with mental well-being, aligning with previous studies linking Committed Action to enhanced quality of life^28^ and mental well-being^60,61^. Increased connection to Values and adherence to value-congruent behaviour resulting from psychedelic experiences have also been tied to better health outcomes^11,13^. While there were no strong direct connections between past psychedelic experiences and Values and Committed Action, the latter showed strong associations with Awareness and Self-as-Context, which, in turn, connected to Acceptance and through it to psychological insights. Due to the cross-sectional nature of the present study, no clear causal direction between psychological insight and Committed Action and Values can be drawn. However, one interpretation suggests that Acceptance functions as a bridging component of psychological flexibility enabling other components, thereby fostering better value orientation and value-congruent behaviour. This proposed sequence aligns with earlier theoretical work on the effects of psilocybin-assisted therapy on psychological flexibility^13^. According to the Accept, Connect, Embody (ACE) model, positive outcomes after psilocybin-assisted therapy result from participants’ acceptance of painful and difficult events that, in turn, leads to an expanded sense of self through awareness of bodily sensations and defusion of rigid mental content. A more expanded sense of self enables the recognition of previously unexplored aspects of the self, which can lead to an increased sense of connectedness to values, meaning, and subsequent value-congruent behaviour^13^. Another interpretation could be that individuals with high trait acceptance and low levels of depression and anxiety experience more psychological insights during psychedelic experiences.

Except for Experiential Avoidance, the results from the community detection algorithm (EGA) in both the Acute and Frequency Models revealed theoretically consistent clustering of both psychological flexibility and inflexibility components, aligning with earlier network and factor analytic research on the multidimensionality of psychological flexibility and psychological inflexibility^23,28,30,62,63^. However, in the present study, nodes reflecting psychological inflexibility were grouped with nodes indexing ill-being in both models, making it challenging to reliably distinguish them from mental ill-being. These findings correspond with earlier findings showing that scales measuring psychological inflexibility, such as the AAQ-II (mainly assessing experiential avoidance), correlate strongly with measures of psychological distress and negative affect^19^. However, due to poor dimensional stability, the results from the EGA in the present study should be interpreted cautiously, and further research is needed to verify these findings.

The finding that Experiential Avoidance was poorly associated with other components of psychological inflexibility and nodes reflecting ill-being contrasts with previous assumptions. Experiential Avoidance, originally considered central to ACT, has historically been synonymous with psychological inflexibility^64^. Most earlier studies on psychological flexibility and psychedelic use have considered low scores on the AAQ-II to indicate greater psychological flexibility^7,8,14,16^. However, the findings from the present study, considering all the psychological flexibility and inflexibility components in the same network, suggest that the variance explained by Experiential Avoidance becomes less prominent when modelled simultaneously with other components. Future studies on the role of psychological flexibility as a therapeutic target in psychedelic therapy should therefore use multidimensional measures of psychological flexibility and inflexibility to better identify crucial interrelations between different components and their direct relevance to psychedelic experiences^24,26^.

### Mediation analyses

The Acute network Model appears to suggest an indirect link between psychological insights and well-being, mediated by psychological flexibility. Indeed, the mediation analysis showed that psychological flexibility mediated the effects of psychological insights on different aspects of well-being and ill-being. More specifically, psychological insights were positively associated with psychological flexibility, and the latter, in turn, was positively associated with mental well-being and peace of mind but negatively associated with symptoms of depression and anxiety. However, given the cross-sectional design of the study, it is impossible to draw any conclusions regarding the causal link between psychological insights and well-being/ill-being.

Surprisingly, experiencing more psychological insights during the psychedelic experience was positively associated with depression and anxiety symptoms. This finding suggests that simply becoming more aware of one’s thoughts and emotions does not necessarily lead to better psychological health but that the addition of, for example, being able to take a flexible and accepting attitude toward these insights is necessary for better health outcomes^10,13,54^. Similar findings have been obtained in mindfulness related research, where increased awareness of difficult feelings can exacerbate psychological problems^65^. However, post hoc analyses showed that the positive relationship between psychological insights and ill-being was explained by the Avoidance and Maladaptive Patterns (AMP) subscale of the PIQ. The subscale reflecting insights into Goals and Adaptive Patterns (GAP) was positively associated with well-being and negatively associated with ill-being. This implies that becoming aware of maladaptive patterns may negatively affect well-being, while awareness of adaptive patterns and possible solutions may mitigate these effects.

Regarding the Frequency Model, when all components of psychological flexibility were aggregated into one single score, mediation analysis showed that the latter mediated the effects of average use on well-being and ill-being. Average use was positively associated with psychological flexibility, which in turn was positively associated with mental well-being and peace of mind but negatively associated with symptoms of depression and anxiety.

### Limitations

This study has several limitations. Firstly, its cross-sectional nature prevents drawing clear causal directions between features of the psychedelic experience, psychological flexibility and well-being/ill-being. Future research employing a longitudinal design with multiple measurement occasions is needed to understand the directionality of the relationship between psychedelic experiences and psychological flexibility. Additionally, underlying traits such as trait acceptance might influence both the psychedelic experience and state measures of psychological flexibility. Future studies could investigate whether high trait acceptance explains the relationship between acute psychedelic experience features such as psychological insights, and psychological flexibility components such as Acceptance.

Secondly, centrality measures in network analysis assume that all relevant nodes are included in a network, limiting their use as clinical indicators^66^. The inclusion of different nodes can yield varied results; however, as a rough summary of complex patterns found in networks, they provide useful information^67^.

Thirdly, retrospective reports of psychedelic experiences may be unreliable due to memory biases. Due to the availability bias, a positive mood at the time of providing the report may lead to a more favourable evaluation of the remembered experience. Another possibility is distorted memory due to the memory–experience gap^68^. More specifically, retrospective reports of affective experiences tend to follow the peak-end rule; we remember the peak affective experience (positive/negative) and the end of the experience and evaluate the whole experience based on either of the two^69^.

Fourthly, the study’s statistical power is worth noting. While no consensus exists on the best method for estimating power in psychological networks, simulations suggest that networks with 11 nodes would achieve adequate power with a sample size of *N* = 450. Although the sample size of the present study is considered adequate, replications with larger samples are warranted.

Lastly, a further limitation is the potential for data quality concerns, such as rushed or inconsistent responses, when utilising online crowdsourcing platforms^70,71^. While data quality checks, like attention checks, are important, this study unfortunately, did not include them. Participants were only screened for consistency in their reports of past psychedelic use, as outlined in the Methods section. The absence of attention checks may have impacted data quality; however, it likely introduced random error rather than systematic bias, thereby weakening the observed effects rather than skewing them.

## Conclusions

Psychological flexibility may underlie the possible long-term mental health benefits of psychedelics. However, most related research has treated psychological flexibility as a unitary construct and conceptualised it as the lack of psychological inflexibility. We examined how the acute features of a meaningful past psychedelic experience and the frequency of psychedelic use are associated with different components of present moment psychological flexibility/inflexibility, as well as with mental well-being/ill-being. According to our network analyses, psychological insights experienced during a meaningful past psychedelic experience were associated with the flexibility component Acceptance. No link was found between the frequency of past psychedelic use and psychological flexibility. Mediation analyses showed that psychological flexibility mediates the effects of psychological insights on well-being and ill-being. Similarly, psychological flexibility mediated the effects of average use on well-being and ill-being but weakly. In contrast to previous research findings, psychological inflexibility was not associated with past psychedelic use according to either the Acute or the Frequency Model, and this construct could not be reliably distinguished from mental ill-being. Taken together, the results suggest that it is mainly the quality and depth of the experience, rather than the quantity or frequency of psychedelic use, that are linked to psychological flexibility, especially Acceptance, as well as psychological flourishing and well-being. Furthermore, given the absence of a direct association between psychological inflexibility and psychedelic use, the results motivate treating psychological flexibility as a multidimensional construct to better understand the relationships between psychedelic use and long-term mental health benefits.

## Electronic supplementary material

Below is the link to the electronic supplementary material.

Supplementary Material 1

## Data Availability

All data used in the analyses is available at the Open Science Framework (https://osf.io/yjs3p/?view_only=d2f99d3432974233af8504cf5198f68f).
