# Supplementary material for: Acceptance as a possible link between past psychedelic experiences and psychological flexibility
Source: Sci Rep. 2024 Oct 16;14:24253. doi: 10.1038/s41598-024-75595-8 (PMC11484703; doi:10.1038/s41598-024-75595-8)
Supplement: Supplementary file 1 — Supplementary Material 1 [file 41598_2024_75595_MOESM1_ESM.docx]

**Appendix**

**Acceptance as a possible link between past psychedelic experience and psychological flexibility**

Andreas Krabbe^a*^, Pilleriin Sikka^b,c,d,e^ and Jussi Jylkkä^a^

^a^Department of Psychology, Åbo Akademi University, Finland
^b^Department of Psychology, University of Turku, Finland
^c^Department of Cognitive Neuroscience and Philosophy, University of Skövde, Sweden
^d^Department of Psychology, Stanford University, CA, USA
^e^Department of Anesthesiology, Perioperative and Pain Medicine, Stanford University, CA, USA

*Corresponding author: Andreas Krabbe
E-mail: [andreas.krabbe@abo.fi](mailto:andreas.krabbe@abo.fi)
Postal address: Åbo Akademi University Arken Tehtaankatu 2, FI-20500 TURKU FINLAND

***Table S1*** *LASSO regularized connectivity coefficients for the Acute Model.*

| **Nodes** | **MEQ** | **PIQ** | **DEP** | **ANX** | **PoMS** | **WEMWBS** | **AC** | **AW** | **SX** | **CT** | **DF** | **CA** | **EA** | **VA** | **LV** | **LA** | **FU** | **IA** |
| --- | --- | --- | --- | --- | --- | --- | --- | --- | --- | --- | --- | --- | --- | --- | --- | --- | --- | --- |
| Mystical Experience (MEQ30) | 0 | 0.47 | 0 | 0 | 0 | 0 | 0 | 0 | 0 | 0 | 0 | 0 | 0 | 0 | 0 | 0 | 0 | 0 |
| Psychological Insight (PIQ) | 0.47 | 0 | 0 | 0 | 0 | 0 | 0.11 | 0 | 0.04 | 0 | 0 | 0 | 0 | 0 | 0 | 0 | 0 | 0 |
| Depression (DEP) | 0 | 0 | 0 | 0.55 | 0 | -0.14 | 0 | 0 | 0 | 0 | 0 | 0 | 0 | 0 | 0 | 0.11 | 0 | 0.09 |
| Anxiety (ANX) | 0 | 0 | 0.55 | 0 | -0.18 | 0 | 0 | 0 | 0 | 0 | 0 | 0 | 0 | 0 | 0 | 0 | 0.08 | 0 |
| Peace of Mind (PoMS) | 0 | 0 | 0 | -0.18 | 0 | 0.51 | 0 | 0 | 0 | 0 | 0.06 | 0 | 0 | 0 | 0 | 0 | -0.12 | 0 |
| Mental Well-being (WEMWBS) | 0 | 0 | -0.14 | 0 | 0.51 | 0 | 0 | 0 | 0 | 0 | 0 | 0.09 | 0 | 0.07 | 0 | 0 | 0 | 0 |
| Acceptance (AC) | 0 | 0.11 | 0 | 0 | 0 | 0 | 0 | 0.19 | 0.13 | 0 | 0 | 0 | 0 | 0 | 0 | 0 | 0 | 0 |
| Awareness (AW) | 0 | 0 | 0 | 0 | 0 | 0 | 0.19 | 0 | 0.18 | 0 | 0 | 0.08 | 0 | 0.19 | 0 | 0 | 0 | 0 |
| Self-as-Context (SX) | 0 | 0 | 0 | 0 | 0 | 0 | 0.13 | 0.19 | 0 | 0 | 0.29 | 0.18 | 0 | 0.16 | 0 | 0 | 0 | 0 |
| Self-as-Content (CT) | 0 | 0 | 0 | 0 | 0 | 0 | 0 | 0 | 0 | 0 | 0 | 0 | 0 | 0 | 0.16 | 0.13 | 0.25 | 0.04 |
| Defusion (DF) | 0 | 0 | 0 | 0 | 0.06 | 0 | 0 | 0 | 0.26 | 0 | 0 | 0 | 0 | 0.12 | 0 | 0 | 0 | 0 |
| Committed Action (CA) | 0 | 0 | 0 | 0 | 0 | 0.09 | 0 | 0.08 | 0.18 | 0 | 0 | 0 | 0 | 0.32 | 0 | 0 | 0 | -0.09 |
| Experiential Avoidance (EA) | 0 | 0 | 0 | 0 | 0 | 0 | 0 | 0 | 0.05 | 0 | 0 | 0 | 0 | 0 | 0 | 0 | 0 | 0 |
| Values (VA) | 0 | 0 | 0 | 0 | 0 | 0.07 | 0 | 0.19 | 0.18 | 0 | 0.12 | 0.32 | 0 | 0 | 0 | 0 | 0 | 0 |
| Lack of Values (LV) | 0 | 0 | 0 | 0 | 0 | 0 | 0 | 0 | 0 | 0.16 | 0 | 0 | 0 | 0 | 0 | 0 | 0.13 | 0.30 |
| Lack of Awareness (LA) | 0 | 0 | 0.11 | 0 | 0 | 0 | 0 | 0 | 0 | 0.13 | 0 | 0 | 0 | 0 | 0 | 0 | 0 | 0 |
| Fusion (FU) | 0 | 0 | 0 | 0.08 | -0.12 | 0 | 0 | 0 | 0 | 0.25 | 0 | 0 | 0 | 0 | 0.13 | 0 | 0 | 0.28 |
| Inaction (IA) | 0 | 0 | 0.09 | 0 | 0 | 0 | 0 | 0 | 0 | 0.04 | 0 | -0.09 | 0 | 0 | 0.30 | 0 | 0.28 | 0 |

*Note.* LASSO regularization penalizes coefficients increasingly as the model complexity grows, causing contraction and specifically reducing smaller coefficients to zero.

| ***Table S2*** *LASSO regularised connectivity coefficients for the Frequency Model* | | | | | | | | | |  |  |  |  |  |  |  |  |  |  |
| --- | --- | --- | --- | --- | --- | --- | --- | --- | --- | --- | --- | --- | --- | --- | --- | --- | --- | --- | --- |
| **Nodes** | **Ave** | **Tim** | **Freq** | **DEP** | **ANX** | **PoM** | **WEMWBS** | **AC** | **AW** | **SX** | **CT** | **DF** | **CA** | **EA** | **VA** | **LV** | **LA** | **FU** | **IA** |
| Average use | 0 | 0 | 0 | 0 | 0 | 0 | 0 | 0 | 0 | 0 | 0 | 0 | 0 | 0 | 0 | 0 | 0 | 0 | 0 |
| Time since last use | 0 | 0 | -0.20 | 0 | 0 | 0 | 0 | 0 | 0 | 0 | 0 | 0 | 0 | 0 | 0 | 0 | 0 | 0 | 0 |
| Cumulative frequency | 0 | -0.20 | 0 | 0 | 0 | 0 | 0 | 0 | 0 | 0 | 0 | 0 | 0 | 0 | 0 | 0 | 0 | 0 | 0 |
| Depression (DEP) | 0 | 0 | 0 | 0 | 0.57 | 0 | -0.14 | 0 | 0 | 0 | 0 | 0 | 0 | 0 | 0 | 0 | 0.09 | 0 | 0.09 |
| Anxiety (ANX) | 0 | 0 | 0 | 0.57 | 0 | -0.19 | 0 | 0 | 0 | 0 | 0 | 0 | 0 | 0 | 0 | 0 | 0 | 0.09 | 0 |
| Peace of Mind (PoMS) | 0 | 0 | 0 | 0 | -0.19 | 0 | 0.51 | 0 | 0 | 0 | 0 | 0.06 | 0 | 0 | 0 | 0 | 0 | -0.12 | 0 |
| Mental Well-being (WEMWBS) | 0 | 0 | 0 | -0.14 | 0 | 0.51 | 0 | 0 | 0 | 0 | 0 | 0 | 0.09 | 0 | 0.07 | 0 | 0 | 0 | 0 |
| Acceptance (AC) | 0 | 0 | 0 | 0 | 0 | 0 | 0 | 0 | 0.20 | 0.14 | 0 | 0 | 0 | 0 | 0 | 0 | 0 | 0 | 0 |
| Awareness (AW) | 0 | 0 | 0 | 0 | 0 | 0 | 0 | 0.20 | 0 | 0.19 | 0 | 0 | 0.09 | 0 | 0.19 | 0 | 0 | 0 | 0 |
| Self-as-Context (SX) | 0 | 0 | 0 | 0 | 0 | 0 | 0 | 0.14 | 0.19 | 0 | 0 | 0.28 | 0.18 | 0 | 0.16 | 0 | 0 | 0 | 0 |
| Self-as-Content (CT) | 0 | 0 | 0 | 0 | 0 | 0 | 0 | 0 | 0 | 0 | 0 | 0 | 0 | 0 | 0 | 0.16 | 0.12 | 0.25 | 0.04 |
| Defusion (DF) | 0 | 0 | 0 | 0 | 0 | 0.06 | 0 | 0 | 0 | 0.28 | 0 | 0 | 0 | 0 | 0.12 | 0 | 0 | 0 | 0 |
| Committed Action (CA) | 0 | 0 | 0 | 0 | 0 | 0 | 0.09 | 0 | 0.09 | 0.18 | 0 | 0 | 0 | 0 | 0.32 | 0 | 0 | 0 | -0.08 |
| Experiential Avoidance (EA) | 0 | 0 | 0 | 0 | 0 | 0 | 0 | 0 | 0 | 0 | 0 | 0 | 0 | 0 | 0 | 0 | 0 | 0 | 0 |
| Values (VA) | 0 | 0 | 0 | 0 | 0 | 0 | 0.07 | 0 | 0.19 | 0.16 | 0 | 0.12 | 0.32 | 0 | 0 | 0 | 0 | 0 | 0 |
| Lack of Values (LV) | 0 | 0 | 0 | 0 | 0 | 0 | 0 | 0 | 0 | 0 | 0.16 | 0 | 0 | 0 | 0 | 0 | 0 | 0.13 | 0.30 |
| Lack of Awareness (LA) | 0 | 0 | 0 | 0.09 | 0 | 0 | 0 | 0 | 0 | 0 | 0.12 | 0 | 0 | 0 | 0 | 0 | 0 | 0 | 0 |
| Fusion (FU) | 0 | 0 | 0 | 0 | 0.09 | -0.12 | 0 | 0 | 0 | 0 | 0.25 | 0 | 0 | 0 | 0 | 0.13 | 0 | 0 | 0.28 |
| Inaction (IA) | 0 | 0 | 0 | 0.09 | 0 | 0 | 0 | 0 | 0 | 0 | 0.04 | 0 | -0.08 | 0 | 0 | 0.30 | 0 | 0.28 | 0 |

*Note.* LASSO regularization penalizes coefficients increasingly as the model complexity grows, causing contraction and specifically reducing smaller coefficients to zero.

***Table S3*** *Raw and standardized centrality estimates for node connectedness in the network.*

*Acute Model*

| **Strength Centrality** | | **Raw** | **Standardized** |
| --- | --- | --- | --- |
| Self-as-Context (SX) | 0.934758775 | | 1.157577749 |
| Depression (DEP) | 0.889266016 | | 0.976656227 |
| Peace of Mind (PoMS) | 0.870207597 | | 0.900862229 |
| Fusion (FU) | 0.854394317 | | 0.837973918 |
| Values (VA) | 0.853878151 | | 0.835921165 |
| Anxiety (ANX) | 0.812502005 | | 0.671371123 |
| Mental Well-being (WEMWBS) | 0.809461502 | | 0.659279256 |
| Inaction (IA) | 0.798490789 | | 0.615649497 |
| Committed Action (CA) | 0.752891887 | | 0.434305854 |
| Acceptance (AC) | 0.683629297 | | -0.819685420 |
| Awareness (AW) | 0.654012681 | | -0.041070169 |
| Lack of Values (LV) | 0.590022645 | | -0.213413731 |
| Psychological Insight (PIQ) | 0.584491071 | | -0.235412411 |
| Self-as-Content (CT) | 0.577792964 | | -0.262050317 |
| Mystical Experience (MEQ30) | 0.471149004 | | -0.686165884 |
| Defusion (DF) | 0.460561651 | | -0.728271047 |
| Lack of Awareness (LA) | 0.234883856 | | -1.625775854 |
| Experiential Avoidance (EA) | 0.000000000 | | -2.559892523 |

***Table S4*** *Raw and standardized centrality estimates for node connectedness in the network.*

*Frequency Model*

| **Strength Centrality** | **Raw** | **Standardized** |
| --- | --- | --- |
| Self-as-Context (SX) | 0.946870 | 1.167300 |
| Depression (DEP) | 0.888220 | 0.984700 |
| Peace of Mind (PoMS) | 0.885100 | 0.975000 |
| Fusion (FU) | 0.858560 | 0.892300 |
| Values (VA) | 0.854700 | 0.880300 |
| Anxiety (ANX) | 0.841980 | 0.840700 |
| Mental Well-being (WEMWBS) | 0.811050 | 0.744400 |
| Inaction (IA) | 0.794780 | 0.693700 |
| Committed Action (CA) | 0.755860 | 0.572500 |
| Awareness (AW) | 0.666480 | 0.294200 |
| Lack of Values (LV) | 0.590020 | 0.056100 |
| Self-as-Content (CT) | 0.569130 | -0.009000 |
| Defusion (DF) | 0.462340 | -0.341600 |
| Acceptance (AC) | 0.338380 | -0.727600 |
| Lack of Awareness (LA) | 0.214750 | -1.112600 |
| Cumulative frequency | 0.195060 | -1.173900 |
| Time since last use | 0.195060 | -1.173900 |
| Average frequency | 0.000000 | -1.781300 |
| Experiential Avoidance (EA) | 0.000000 | -1.781300 |

***Table S5*** *bootstrapped EGA*

| Frequency of dimensions across all EGA bootstrap replicate samples for the Acute Model | | |
| --- | --- | --- |
| # of dimensions | Frequency |  |
| 3 | 0.036 |  |
| 4 | 0.886 |  |
| 5 | 0.076 |  |
| 6 | 0.002 |  |

***Table S6*** *bootstrapped EGA*

| Frequency of dimensions across all EGA bootstrap replicate samples for the Frequency Model | | |
| --- | --- | --- |
| # of dimensions | Frequency |  |
| 3 | 0.012 |  |
| 4 | 0.853 |  |
| 5 | 0.118 |  |
| 6 | 0.017 |  |

***Figure S1*** *The bootstrapped edge weights for the Acute Model*


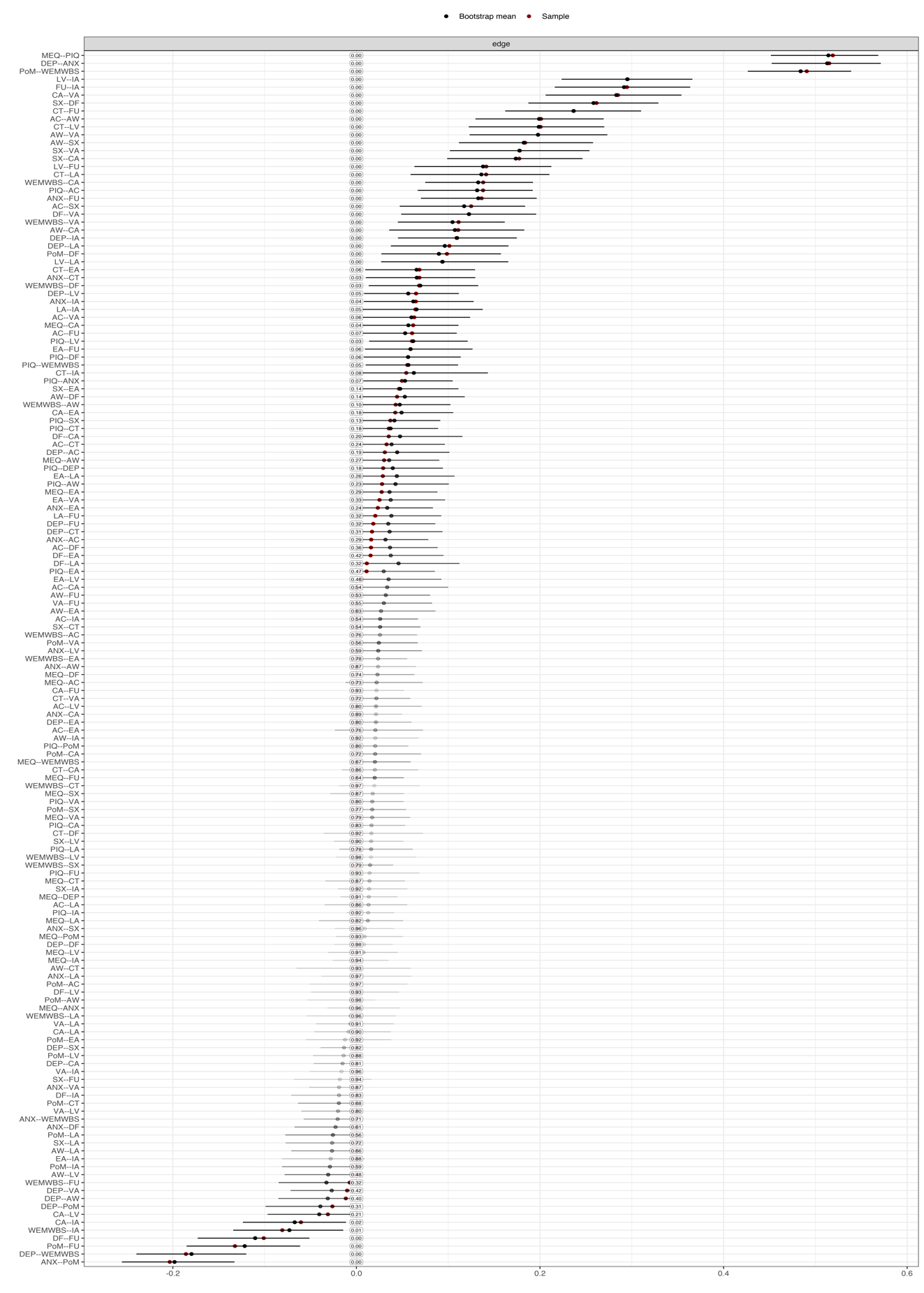
*Note.* All edges from the network displayed on the Y-axis. X-axis represents strength of the edges. The proportion of bootstrapped samples that return an edge-weight estimate of zero are displayed in boxes stacked vertically at zero on the x-axis. *ANX = anxiety, DEP = depression, FU = fusion, CT = self-as-content, LA = lack of awareness, IA = inaction, LV = lack of values, EA = experiential avoidance, PoM = peace of mind, WEMWBS = mental well-being, CA = committed action, DF = defusion, VA = values, SX = self-as-context, AW = awareness, AC = acceptance, PIQ = psychological insight, MEQ = mystical-type experience.*

***Figure S2*** *The bootstrapped edge weights for the Frequency Model*

***
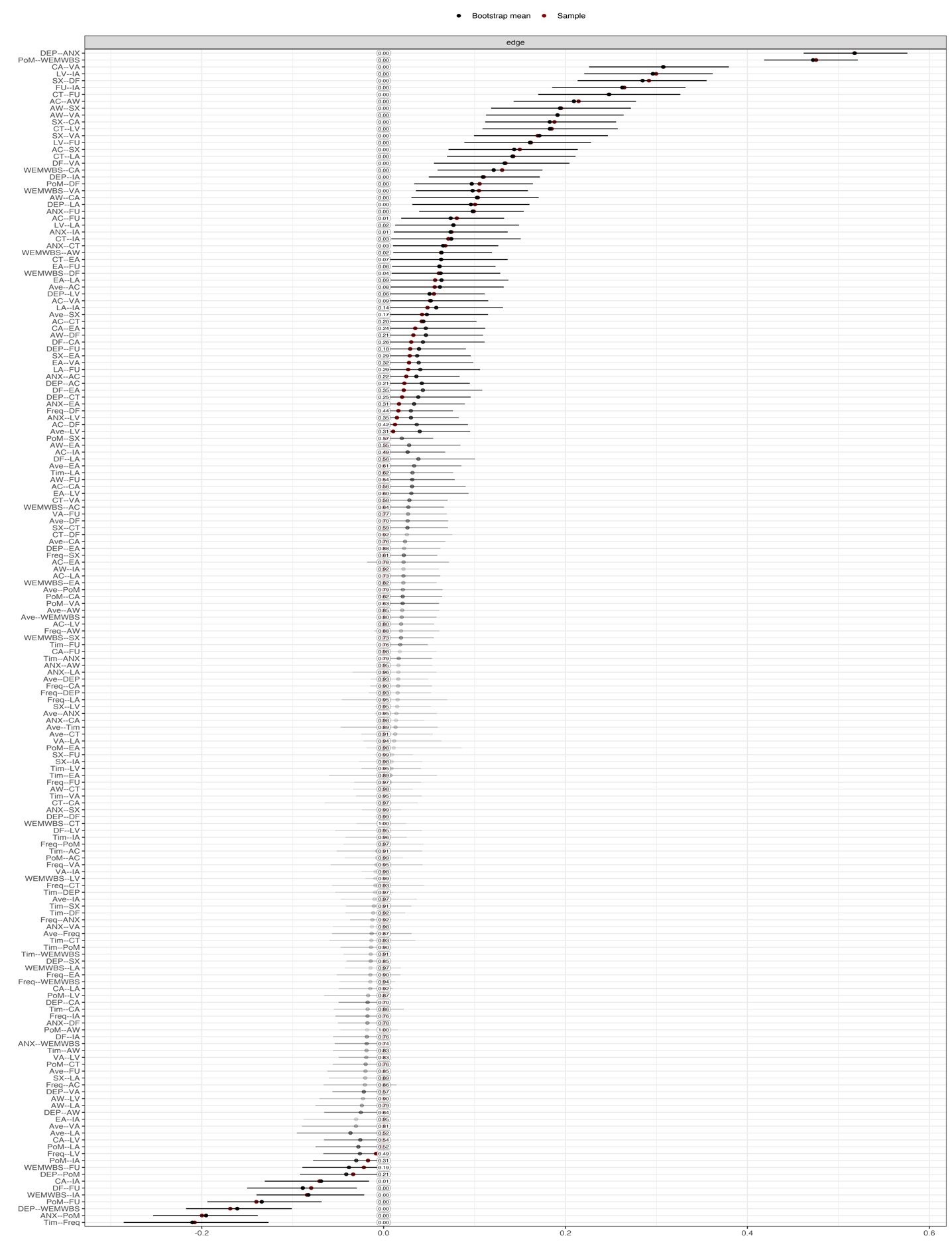
****Note.* All edges from the network displayed on the Y-axis. X-axis represents strength of the edges. The proportion of bootstrapped samples that return an edge-weight estimate of zero are displayed in boxes stacked vertically at zero on the x-axis. *ANX = anxiety, DEP = depression, FU = fusion, CT = self-as-content, LA = lack of awareness, IA = inaction, LV = lack of values, EA = experiential avoidance, PoM = peace of mind, WEMWBS = mental well-being, CA = committed action, DF = defusion, VA = values, SX = self-as-context, AW = awareness, AC = acceptance, Ave = average frequency of psychedelic use, Tim = Time since last use, Freq = Cumulative frequency.*

***Figure S3*** *Case-drop bootstrap plot for the reported centrality indices for the Acute Model.*


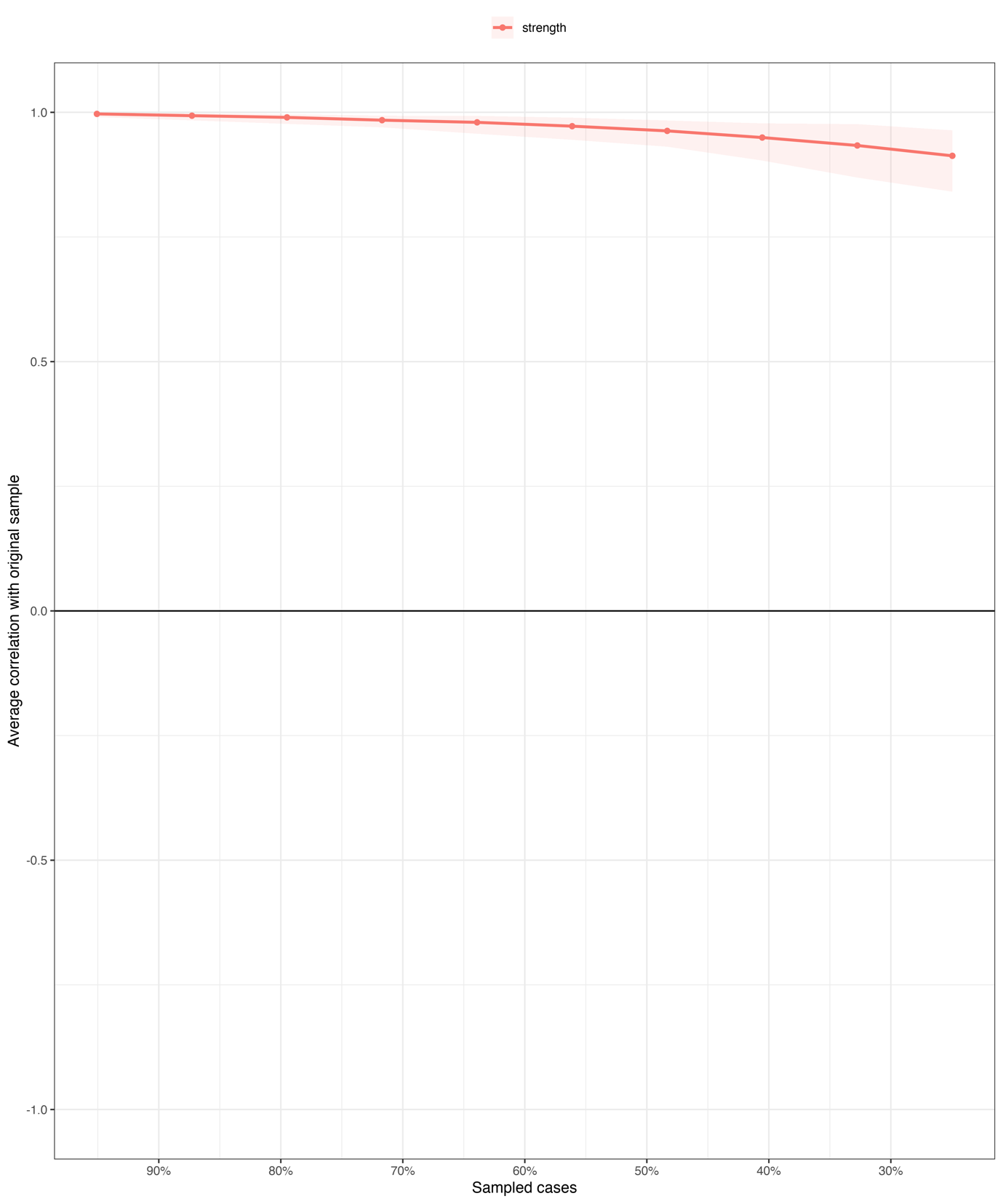


***Figure S4*** *Case-drop bootstrap plot for the reported centrality indices for the Frequency Model.*

***
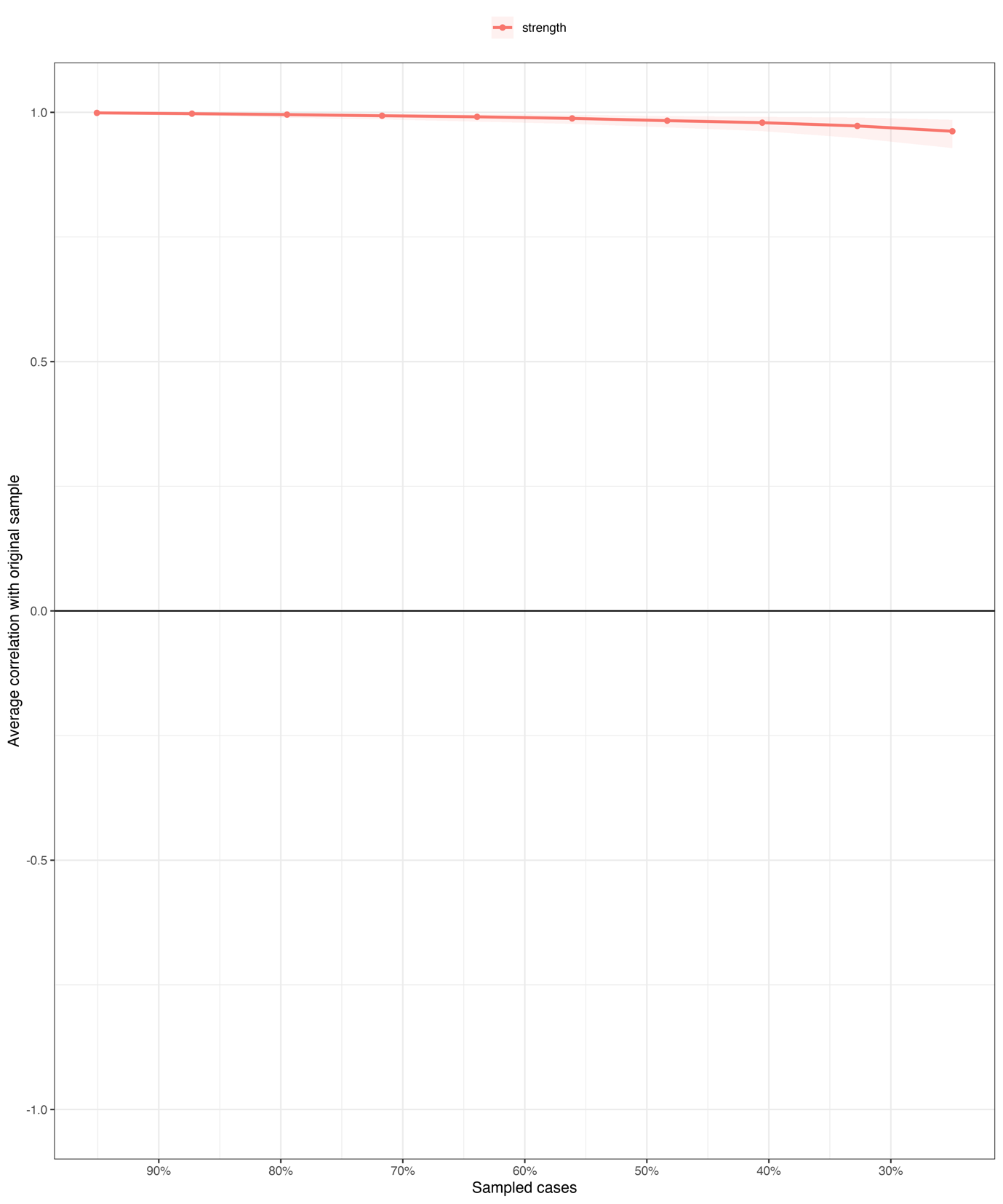
***

***Figure S5*** *Stability of empirical EGA communities for the Acute Model.*


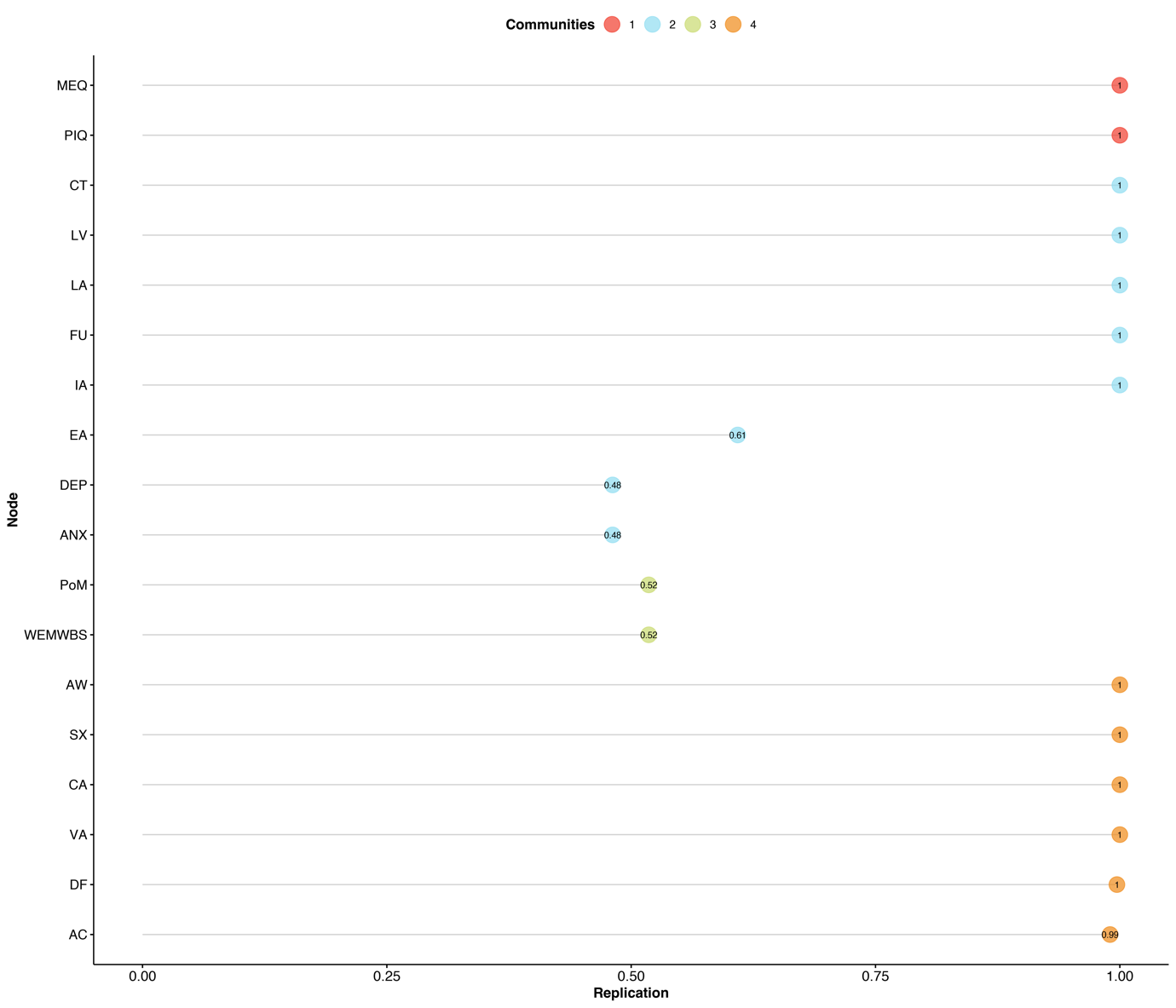
*Note.* *ANX = anxiety, DEP = depression, FU = fusion, CT = self-as-content, LA = lack of awareness, IA = inaction, LV = lack of values, EA = experiential avoidance, PoM = peace of mind, WEMWBS = mental well-being, CA = committed action, DF = defusion, VA = values, SX = self-as-context, AW = awareness, AC = acceptance, PIQ = psychological insight, MEQ = mystical-type experience.*

***Figure S6*** *Stability of empirical EGA communities for the Frequency Model.*


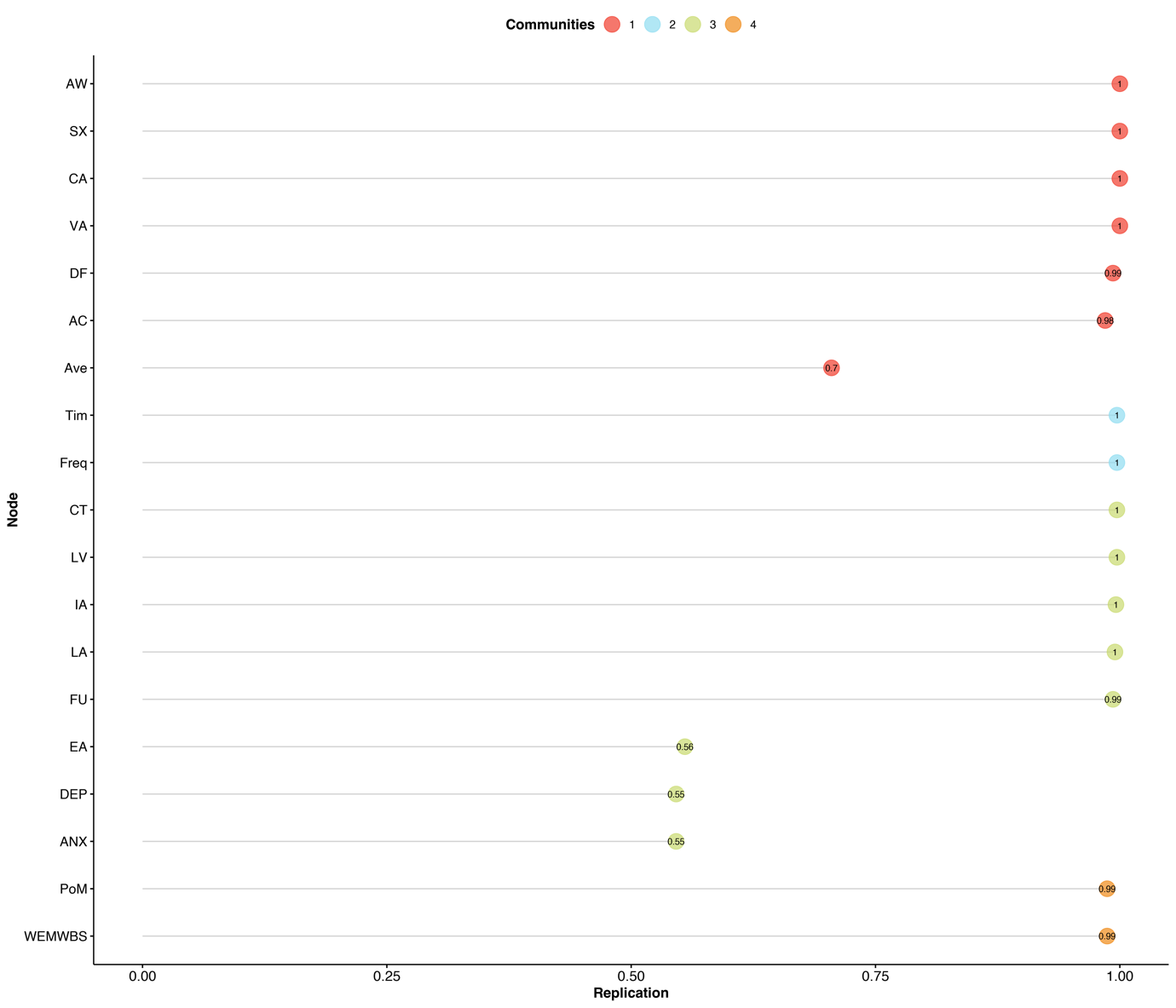


*ANX = anxiety, DEP = depression, FU = fusion, CT = self-as-content, LA = lack of awareness, IA = inaction, LV = lack of values, EA = experiential avoidance, PoM = peace of mind, WEMWBS = mental well-being, CA = committed action, DF = defusion, VA = values, SX = self-as-context, AW = awareness, AC = acceptance, Ave = average frequency of psychedelic use, Tim = Time since last use, Freq = Cumulative frequency.*
